# Accurate and Rapid Detection of Peritoneal Metastasis from Gastric Cancer by AI‐Assisted Stimulated Raman Molecular Cytology

**DOI:** 10.1002/advs.202300961

**Published:** 2023-04-28

**Authors:** Xun Chen, Zhouqiao Wu, Yexuan He, Zhe Hao, Qi Wang, Keji Zhou, Wanhui Zhou, Pu Wang, Fei Shan, Zhongwu Li, Jiafu Ji, Yubo Fan, Ziyu Li, Shuhua Yue

**Affiliations:** ^1^ Key Laboratory of Biomechanics and Mechanobiology Ministry of Education Institute of Medical Photonics Beijing Advanced Innovation Center for Biomedical Engineering School of Biological Science and Medical Engineering Beihang University 100191 Beijing China; ^2^ Gastrointestinal Cancer Center Key Laboratory of Carcinogenesis and Translational Research (Ministry of Education) Peking University Cancer Hospital and Institute 100142 Beijing China; ^3^ School of Engineering Medicine Beihang University 100191 Beijing China; ^4^ Key Laboratory of Carcinogenesis and Translational Research (Ministry of Education) Department of Pathology Peking University Cancer Hospital and Institute 100142 Beijing China

**Keywords:** cytology, deep learning, gastric cancer, metastasis, stimulated Raman scattering

## Abstract

Peritoneal metastasis (PM) is the mostcommon form of distant metastasis and one of the leading causes of death in gastriccancer (GC). For locally advanced GC, clinical guidelines recommend peritoneal lavage cytology for intraoperative PM detection. Unfortunately, current peritoneal lavage cytology is limited by low sensitivity (<60%). Here the authors established the stimulated Raman molecular cytology (SRMC), a chemical microscopy‐based intelligent cytology. The authors firstly imaged 53 951 exfoliated cells in ascites obtained from 80 GC patients (27 PM positive, 53 PM negative). Then, the authors revealed 12 single cell features of morphology and composition that are significantly different between PM positive and negative specimens, including cellular area, lipid protein ratio, etc. Importantly, the authors developed a single cell phenotyping algorithm to further transform the above raw features to feature matrix. Such matrix is crucial to identify the significant marker cell cluster, the divergence of which is finally used to differentiate the PM positive and negative. Compared with histopathology, the gold standard of PM detection, their SRMC method could reach 81.5% sensitivity, 84.9% specificity, and the AUC of 0.85, within 20 minutes for each patient. Together, their SRMC method shows great potential for accurate and rapid detection of PM from GC.

## Graphical abstract

For locally advanced gastric cancer, clinical guidelines recommend peritoneal lavage cytology for intraoperative PM detection. Unfortunately, current peritoneal lavage cytology is limited by low sensitivity (<60%). Here the stimulated Raman molecular cytology (SRMC), a chemical microscopy‐based intelligent cytology, is established. This SRMC method assisted by machine learning classifiers could reach 81.5% sensitivity, 84.9% specificity within 20 min for each patient.

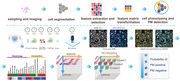

## 1 Introduction

Gastric cancer (GC) ranks third in cancer‐related deaths worldwide.^[^ ^1^ ^]^ The leading cause of death in GC is metastasis, in which most common form (50–60%) is peritoneal metastasis (PM).^[^ ^2^, ^3^ ^]^ Since GC patients with or without PM receive substantially different treatment strategies, including surgery and neoadjuvant therapies, accurate PM diagnosis is of great clinical significance for treatment and prognosis.^[^ ^4^, ^5^, ^6^ ^]^

The gold standard for PM diagnosis in GC is histopathological examination of peritoneal tissue biopsy, which is invasive and thus suggested to be obtained intraoperatively under laparoscopy. The procedure of histopathological diagnosis of PM is time‐consuming and cannot provide results in a timely manner during surgery. Alternatively, preoperative computed tomography offers a way to detect PM noninvasively, but it is not sensitive enough, which leads to fatal false negatives.^[^ ^7^, ^8^ ^]^ Peritoneal lavage cytology, developed based on the theory that PM of GC is induced by colonization of exfoliated GC cells in the peritoneum, has been shown to be more sensitive than computed tomography, and more efficient and less invasive than histopathology.^[^ ^9^ ^]^ Owing to these advantages, clinical guidelines in various countries have recommended peritoneal lavage cytology for patients with locally advanced GC during surgery,^[^ ^10^, ^11^, ^12^ ^]^ which can be potentially extended to preoperative diagnosis and postoperative follow‐up.^[^ ^13^ ^]^ Nevertheless, the accuracy of conventional peritoneal lavage cytology for PM diagnosis is still limited, with sensitivity even lower than 60%,^[^ ^14^, ^15^, ^16^, ^17^, ^18^ ^]^ and highly relies on pathologists.^[^ ^19^ ^]^

To increase the detection accuracy of GC cells in ascites, several biochemical and molecular biology methods have been developed, such as enzyme‐linked immunosorbent assay (ELISA)^[^ ^20^ ^]^ or flow cytometry‐based detection of specific proteins,^[^ ^21^, ^22^ ^]^ reverse transcriptase polymerase chain reaction (RT‐PCR) or fluorescence in situ hybridization (FISH)‐based detection of specific genes.^[^ ^23^, ^24^, ^25^, ^26^, ^27^, ^28^ ^]^ However, these methods are too time‐consuming for intraoperative detection. More recently, a label‐free optically induced electrokinetics microfluidic method was developed to efficiently separate GC cells from ascites of six patients with purity up to 71%,^[^ ^21^ ^]^ but its performance on PM detection was not shown. Therefore, a new cytology method with both high accuracy and efficiency is urgently needed for detection of PM in GC.

Besides gene and protein expression, altered cell metabolism has been recognized as a hallmark of human cancers.^[^ ^29^, ^30^ ^]^ Cancer cells dysregulate metabolic pathways by high rates of lipid synthesis to support rapid growth.^[^ ^29^, ^30^ ^]^ GC cells have been found to accelerate lipid synthesis and reduce lipid hydrolysis,^[^ ^31^, ^32^ ^]^ which leads to increased accumulation of excessive lipids in lipid droplets (LDs).^[^ ^31^, ^32^ ^]^ More importantly, dysregulated lipid metabolism has been shown to promote cancer metastasis,^[^ ^33^ ^]^ including PM.^[^ ^34^ ^]^ Particularly, as discovered by metabolomics, a variety of lipid molecules, such as triglycerides, sterols, fatty acids, could be used as biomarkers for PM of GC.^[^ ^35^ ^]^

For single cell molecular analysis, Raman spectroscopy is a commonly used label‐free method. Several studies have demonstrated the potential of Raman spectroscopy in cytopathology for diagnosis of cancers, including cervical cancer, lung cancer, and oral cancer.^[^ ^36^, ^37^, ^38^ ^]^ AI algorithms improved the robustness of Raman spectroscopy for high‐precision cancer diagnosis.^[^ ^39^ ^]^ However, due to the weak spontaneous Raman signals, Raman spectroscopy‐based cytology took up to 8 h for a complete analysis without spatial information. With remarkably boosted Raman signals, simulated Raman scattering (SRS) microscopy is a desirable method of label‐free and high speed molecular imaging at the single cell level.^[^ ^40^, ^41^ ^]^ In recent years, SRS microscopy with Raman tags^[^ ^42^ ^]^ has been widely used in the study of cancer metabolism^[^ ^43^ ^]^ and diagnosis.^[^ ^44^ ^]^ For instance, Ji et al. for the first time employed two‐color SRS microscopy to achieve virtual H&E staining, that is stimulated Raman histology (SRH),^[^ ^45^, ^46^ ^]^ and later on Hollon et al. demonstrated deep learning‐based SRH could realize intraoperative brain tumor diagnosis.^[^ ^47^ ^]^ The SRH method has been applied in diagnosis of multiple human cancers.^[^ ^48^, ^49^, ^50^, ^51^, ^52^ ^]^ Unfortunately, because cytology is not the gold standard for PM diagnosis in GC, the concept of SRH that primarily depends on morphology cannot be transferred to cytology‐based PM diagnosis. Taken together, future cytology method of PM detection in GC probably requires an efficient acquisition of the information regarding both cellular morphology and composition, which is readily accessible by SRS microscopy.

In this study, we developed the stimulated Raman molecular cytology (SRMC), an SRS microscopy‐based intelligent cytology method for diagnosis of PM from GC. By incorporating deep learning based single cell segmentation algorithms with three‐color SRS imaging at the Raman bands corresponding to DNA, protein, and lipid, we first extracted 19 single cell features of morphology and composition from the exfoliated cells of ascites. Among them, 12 features including cellular area, lipid protein ratio, and lipid droplets number, etc. of exfoliated cells (*N* = 53 951 cells) were significantly different between PM positive and negative. Then, by newly developed hybrid K‐means cell clustering and principal component analysis (PCA) algorithm (K‐PCA), the differential raw features were dimensionally reduced and transformed to feature matrix in clustered latent space, which allowed us to find out the significant marker cell population. Finally, the feature divergence of significant marker cells was used to differentiate the PM positive and negative. A panel of machine learning classifiers, such as support vector machine (SVM), linear discriminate analysis (LDA), and logistic regression (LR), were used to train the PM diagnostic model with the feature matrix and the ground truth of PM results as inputs. By cross‐validation, the sensitivity and specificity of our SRMC method for PM detection were 81.5% and 84.9% respectively (*n* = 80 patients) within 20 min. Particularly, by providing composition information, the sensitivity of PM detection by the SRMC significantly improved from 59.25% to 81.5%, suggesting that both cellular morphology and composition are essential for accurate diagnosis. Collectively, our SRMC method may open up new opportunities for accurate and rapid detection of PM in GC with minimal invasion.

## 2 Results

### 2.1 Workflow of Stimulated Raman Molecular Cytology

**Figure 1:**
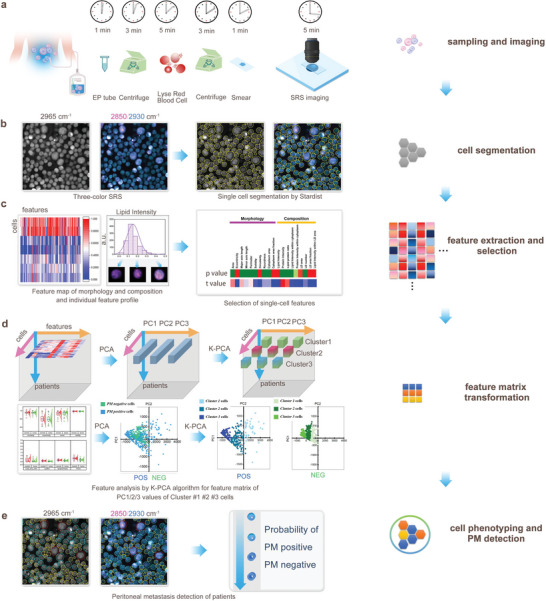
Workflow of stimulated Raman molecular cytology (SRMC). a) Sample preparation and SRS imaging. b) Single cell segmentation based on three‐color SRS images. c) Single cell feature extraction and selection. d) Feature matrix transformation from raw features by K‐PCA algorithm. e) Cell phenotyping by K‐PCA, and PM positive and negative detection by machine learning classifiers.

As shown in **Figure** **1**, the workflow of SRMC is described. 1) First, the three‐color SRS imaging were performed on the exfoliated cells collected from the ascites of patients with locally advanced GC (Figure 1a). Specifically, we acquired the SRS images of the Raman band for CH_2_ stretching in lipids around 2850 cm^−1^, the Raman band for CH_3_ stretching in proteins around 2930 cm^−1^ and the Raman band for CH_3_ antisymmetric stretching in DNA around 2965 cm^−1^, respectively. 2) Second, individual exfoliated cells were segmented based on the SRS images of DNA Raman band, by using deep learning based Stardist model (Figure 1b). 3) Third, a variety of features on cellular morphology and composition of single exfoliated cells for each patient were extracted. The features with statistically significant difference between PM positive and PM negative specimens were further selected to create the raw feature map (Figure 1c). 4) Fourth, the raw feature map then went through dimensionality reduction by principal component analysis (PCA) to get the latent space, including PC1, PC2 components etc. for each cell. The principal component was then transformed by K‐means clustering to get transformed feature matrix in clustered latent space of PCA, such as PC1‐center value, PC2‐center value, etc. that were the medians of the corresponding PC values for each cell cluster (Figure 1d). Here, we defined this hybrid PCA and K‐means clustering algorithm as K‐PCA, in which the PCA filtered out features with low standard deviation and K‐means clustering built features spacing between clusters. The transformed feature matrix by K‐PCA represented divergence of features for each cell cluster. The PC1 versus PC2 plot showed feature matrix of PC 1/2/3 values of Cluster #1/#2/#3 cells from PM positive and PM negative specimens (Figure 1d). 5) Finally, the transformed feature matrix was used to train machine learning‐based PM diagnostic models with histopathology as the ground truth. The performance of our SRMC method in PM detection, such as sensitivity, specificity, and the area under receiver operating characteristic (ROC) curve, were further evaluated by leave‐one‐out cross‐validation (Figure 1e). The detailed procedures were described in Section 4.

### 2.2 Single Cell Segmentation and Feature Extraction

By using deep learning Stardist network (Figure S1a, Supporting Information), we achieved single cell segmentation based on the SRS image of DNA Raman band around 2965 cm^−1^ with great performance (Dice parameter 0.89, IoU 0.81, and RRSE 2.3%), which was significantly better compared to conventional watershed or flood fill methods (Figure S1b,c, Supporting Information). As shown in Figure S2, Supporting Information, the feature extraction workflow was described in detail. The morphology features, including cellular area, minor axis length, major axis length, circularity, roundness, eccentricity, perimeter, and solidity, were extracted based on cell masks obtained from the 2965 cm^−1^ channel. The composition features, including lipid intensity, protein intensity, and lipid protein ratio, were extracted based on the intensity values obtained from 2850 cm^−1^ and 2930 cm^−1^ channels within the cell masks. According to the 2850 cm^−1^ channel, we recognized LDs by adaptive thresholding, which allowed quantification of LD area, LD number, LD area fraction, and lipid intensity within LD area. Based on calculations of 2850 cm^−1^ and 2965 cm^−1^ channels, nuclei could be identified by adaptive thresholding, which permit measurements of nucleus area, cytoplasm area, and cytoplasm area fraction. Then, the lipid intensity and protein intensity within cytoplasm could be quantified. The detailed procedures of feature extraction were described in Section 4.

### 2.3 SRMC of Gastric Cell Lines

Considering that peritoneal lavage exfoliated cells in GC patients are primarily composed of benign gastric epithelial cells, mesothelial cells, and malignant GC cells, we performed SRMC on three presentative cell lines, including gastric epithelium GES‐1, differentiated carcinoma SNU‐16, and mesothelium HMrSV5. As shown in Figure S3a,b, Supporting Information, we first extracted three features of cellular morphology (area, circularity, and roundness) and four features of chemical composition (lipid intensity, protein intensity, lipid protein ratio, and LDs area fraction) for individual cells. Among these features, the lipid intensity was significantly greater in malignant cancer cells compared to normal epithelial and mesothelial cells, suggesting that malignant GC cells might accumulate more lipids than other types of exfoliated cells. In addition, the correlation coefficients were relatively high within morphology features (for instance, roundness, and circularity) or within composition features (for instance, lipid intensity and protein intensity), but were relatively low between morphology and composition features (Figure S3c, Supporting Information). By using LDA algorithm based on all the features, we could differentiate the GC cells from normal epithelium and mesothelium with the accuracy of 98.36% and 100%, respectively (Figure S3d, Supporting Information). These results from cell lines demonstrated the potential of the SRMC method for GC cell detection by integrating single‐cell morphology and composition features.

### 2.4 Differential Features of Cellular Morphology and Composition between PM Positive and Negative Specimens

We quantitatively characterized 53 951 individual exfoliated cells in ascites obtained from 80 GC patients (27 PM positive, 53 PM negative). As shown in Figure S4, Supporting Information, 10 features of cellular morphology and 9 features of chemical composition were extracted. Student's *t*‐test was used for comparisons between groups. The *t*‐value is to quantify the difference between two group means. A larger *t*‐value shows that the difference between the group means is greater than the standard error, indicating a more significant difference between two groups. The *p*‐value is the conditional probability of obtaining at least as *t*‐value from data distribution of two groups, and *p*‐value < 0.05 was considered statistically significant. Among all the features, 7 morphology features, including cellular area, major axis length, minor axis length, perimeter, solidity, cytoplasm area, and roundness, and 5 composition features, including protein intensity, lipid protein ratio, and lipid intensity within cytoplasm, protein intensity within cytoplasm, and LD number were significantly different between PM positive and PM negative specimens. As shown in the *t*‐value hot map, the top three differential features were protein intensity, lipid protein ratio, and cellular area. Nevertheless, the differences in composition and morphology features between PM positive and negative specimens were not very evident, which was likely due to the existence of heterogeneous populations in each specimen. Therefore, we proposed to further analyze the features by PCA and clustering algorithms.

### 2.5 PM Related Significant Marker Cell Population Identified by K‐PCA Algorithm

Since exfoliated cells in PM positive specimens are composed of both tumor cells and normal cells (primarily mesothelial cells), it is necessary to identify the significant marker cell population with specific signatures for PM diagnosis. First, the raw feature dataset was processed with dimensionality reduction by PCA, which produced the primary PC1 and PC2 components accounting for >90% of total features. Then, the feature components were transformed by K‐means clustering to get transformed feature matrix of clustered latent space, including Cluster1‐PC1, Cluster1‐PC2, Cluster2‐PC1, Cluster2‐PC2, Cluster3‐PC1, Cluster3‐PC2, etc. Cluster‐number defined the number of cells for each cluster. The principal components of cell features indicated three nearest neighbors by assessing the lowest neighbor distance. Therefore, we used three clusters for cell phenotyping. Without dimensionality reduction, the raw features were related to PM results with low correlation coefficient (*R* ^2^ < 0.3) (Figure S5 and Table S1, Supporting Information). With dimensionality reduction by K‐PCA, the Cluster1‐PC1 component was related to PM results with improved correlation coefficient (*R* ^2^ = 0.67) (Figure S5, Supporting Information). The details of cell numbers and PC values for each cluster of 80 patients are shown in Table S2, Supporting Information. For instance, Patient ID NEG#1(PM POS) has 29 Cluster1 cells, 180 Cluster2 cells, and 306 Cluster3 cells respectively, and Cluster1‐PC1 and Cluster1‐PC2 are 1737.094 and 173.373 respectively. Patient ID POS#1(PM POS) has 15 Cluster1 cells, 277 Cluster2 cells, and 304 Cluster3 cells respectively, and Cluster1‐PC1 and Cluster1‐PC2 are 1351.002 and 45.233 respectively. Patient ID NEG#2 (PM NEG) has 51 Cluster1 cells, 296 Cluster2 cells, and 245 Cluster3 cells respectively, and Cluster1‐PC1 and Cluster1‐PC2 are 844.614 and −7.123 respectively. Based on the hot maps of raw features and feature matrix before and after K‐PCA (*n* = 80 patients), Cluster1 number and Cluster1‐PC1 were obviously different between PM positive and negative (Figure S5, Supporting Information). PC1‐values of PM positive specimens were significantly higher than those of PM negative specimens.

**Figure 2:**
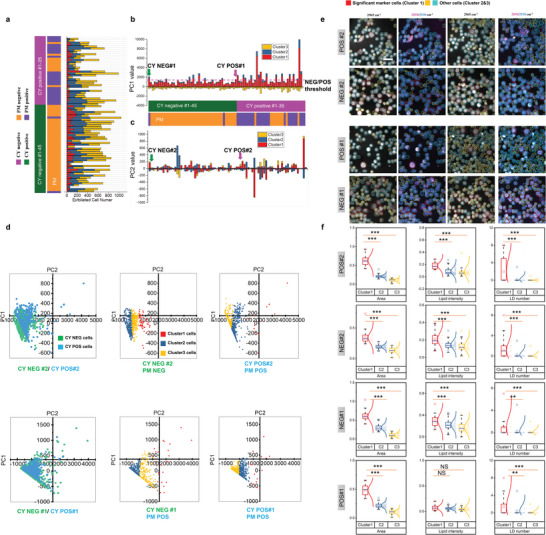
Feature matrix transformation by K‐PCA algorithm. a) Exfoliated cell numbers of 80 patients by clustering. b) PC1 values of clusters (Cluster 1, 2, 3) of 80 patients with a threshold of Cluster1‐PC1. c) PC2 values of clusters (Cluster 1, 2, 3) of 80 patients. d) Plot of PC1 versus PC2 for representative CY NEG#1 (diagnosed with PM positive), POS#1 (diagnosed with PM positive) and CY NEG#2 (diagnosed with PM negative), POS#2 (diagnosed with PM positive) before and after K‐means clustering. e) Representative SRS images of CY NEG/POS#1 and CY NEG/POS#2. f) Quantitative comparisons of features (LD number, lipid intensity, cellular area) among clusters in CY NEG/POS#1 and CY NEG/POS#2. The box and whisker plots represent median values (center lines), mean values (horizontal bars), minimum and maximum (outliers), 25th to 75th percentiles (box edges) and 1.5× interquartile range (whiskers), with all points plotted. ***p < 0.0005, **p < 0.005, *p < 0.05, ns: no significant difference.

**Figure 3:**
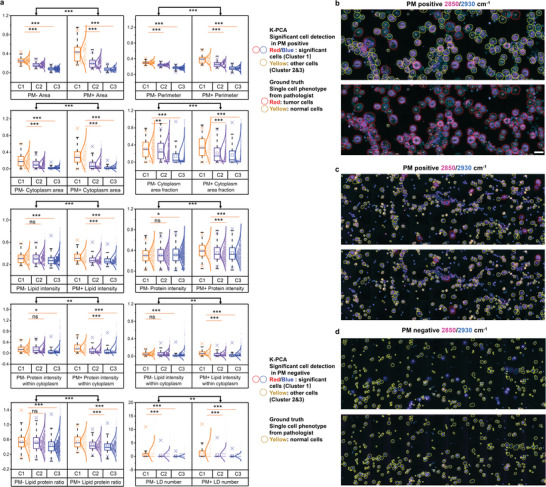
Significant marker cell identification by K‐PCA algorithm. a) Quantitative comparisons of features among clusters (Cluster 1: significant marker cells; Cluster 2 and 3: other cells) between all PM positive and negative specimens. The box and whisker plots represent median values (center lines), mean values (horizontal bars), minimum and maximum (outliers), 25th to 75th percentiles (box edges) and 1.5× interquartile range (whiskers), with all points plotted. ***p < 0.0005, **p < 0.005, *p < 0.05, ns: no significant difference. b–d) Representative SRS images (2850 cm^−1^ and 2930 cm^−1^) with clusters identified by K‐PCA algorithm and tumor/normal cells identified by pathologists using the conventional cytology of the same specimen as reference, for PM positive specimen with high percentage of tumor cells, PM positive specimen with low percentage of tumor cells, and typical PM negative specimen without tumor cells. Scale bar, 20 µm.

We analyzed the cell number (**Figure** **2**a), Cluster1,2,3‐PC1 (Figure 2b) and Cluster1,2,3‐PC2 (Figure 2c) of 80 patients (27 PM positive, 53 PM negative). Notably, the Cluster1‐PC1 values were more closely correlated with PM results than conventional cytology (CY) results of the same patients. A threshold of Cluster1‐PC1 was obtained based on ROC curve (Figure S5, Supporting Information). Specifically, there appeared to be a threshold (threshold = 1380) of Cluster1‐PC1 for determining PM NEG/POS by the ROC curves to get the best diagnostic sensitivity and specificity (AUC = 0.785) (Figure 2b). The Cluster1‐PC1 was higher than the threshold in CY NEG#1 patient diagnosed with PM positive (Figure 2b), but lower than the threshold in CY NEG#2 patient diagnosed with PM negative (Figure 2c). By comparing the K‐PCA latent spaces from two representative pairs of patients, as shown in Figure 2d, the principal components of Cluster1 from PM positive specimens dispersed to much greater distance within clusters relative to those from PM negative specimens, suggesting that *Cluster1* probably contained features closely related to PM. Thus, Cluster1 was defined as the significant marker cell population as shown in Figure 2e. The PC1 versus PC2 plot of CY NEG#1 (diagnosed with PM positive) was indeed similar to those of PM positive. After cell clustering, we could characterize the morphology and composition features for different clusters of cells. As shown in Figure 2f and Figure S6, Supporting Information, morphology and composition features, such as cellular area, lipid intensity and LD number, were significantly different among different clusters. As shown in **Figure** **3**, we analyzed differences of features between all the PM positive and negative specimens. The overall 10 features such as cellular area, perimeters, cytoplasm area, cytoplasm area fraction, lipid intensity, protein intensity, lipid protein ratio, protein intensity within cytoplasm, lipid intensity within cytoplasm and LD number were significantly different between PM positive and negative after K‐PCA (Figure 3a). In terms of different clusters, the features, such as area, perimeter, cytoplasm area fraction, cytoplasm area and LD number, were significantly different between significant marker cells (Cluster 1) and other cells (Cluster 2 and 3) (Figure 3a). These results together indicate that our K‐PCA algorithm has a potential to accurately evaluate PM by introducing more information on composition besides morphology used in conventional cytology.

Moreover, we explored the relationship between significant marker cells and tumor or normal cells. The SRS images of exfoliated cells were first stitched into larger scale (Figure S7, Supporting Information) that could be comparable with the H&E cytological images of the same locations in situ. Based on the labeling of normal and tumor cells on the cytological images by pathologists, we then integrated machine learning (ML) methods with PCA, called ML‐PCA here, to differentiate tumor cells from normal cells. The accuracy, sensitivity, specificity and the area under ROC curve (AUC) of ML‐PCA methods were 93.8%, 94.1%, 93.6%, and 0.98 for SVM based ML‐PCA, and 90.5%, 92.0%, 90.1%, and 0.96 for LDA based ML‐PCA (Figure S8, Supporting Information). As shown in Figure 3b–d and Figure S9, Supporting Information, the significant marker cell population in PM positive was primarily included in the tumor cell population, especially for PM positive specimens with high percentage of tumor cells. In the meanwhile, the significant marker cell population in PM negative specimen belonged to the normal cell population. These findings suggest that the significant marker cell population possibly contains features in which the divergence highly correlates with PM. The differences of features between significant marker cells and other cells identified by K‐PCA were more evident than the differences of features between tumor cells and normal cells identified by ML‐PCA, especially for PM positive specimens with high percentage of tumor cells (Figure S10, Supporting Information). Collectively, in SRMC method, we do not need to follow conventional cytology to capture all tumor cells, but rather use K‐PCA algorithm to identify the significant marker cells with specific morphology and composition features, in which the divergence is likely related to PM.

### 2.6 Demonstration of PM Detection by SRMC

**Figure 4:**
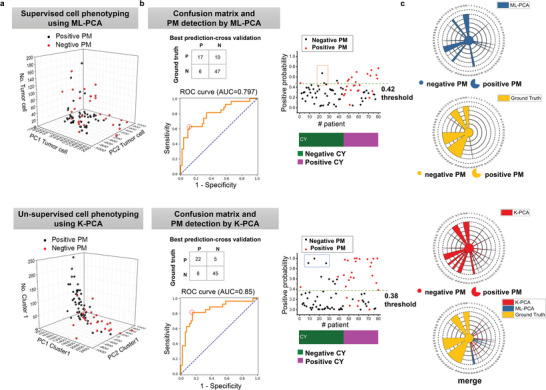
PM detection by SRMC using machine learning classifiers. a) Upper: Input feature matrix (PC1, PC2, cell number of tumor cell) of PM positive and negative specimens by the ML‐PCA method (SVM). Down: Input feature matrix (PC1, PC2, cell number of Cluster 1) of PM positive and negative specimens by the K‐PCA method. b) Upper: Confusion matrix, positive probability with cross‐validation, and ROC curve by the ML‐PCA method (SVM) with a threshold to get best sensitivity and specificity. Down: Confusion matrix, positive probability with cross‐validation, and ROC curve by the K‐PCA method with a threshold to get best sensitivity and specificity. c) Upper: Final detection results of 80 patients by ML‐PCA (SVM) compared with ground truth. Down: Final detection results of 80 patients by K‐PCA compared with ground truth.

The transformed feature matrix was further used as input features in machine learning models for PM prediction. As shown in **Figure** **4**a, the feature matrix obtained by K‐PCA (Cluster1‐PC1, Cluster1‐PC2, Cluster1‐number etc.) could differentiate PM positive and negative more clearly than the feature matrix obtained by ML‐PCA. The feature matrix was then trained and validated with the ground truths of PM diagnosis by using machine learning methods, including SVM, LDA, and LR. With leave‐one‐out cross‐validation, PM positive probability of each patient, ROC curve, and confusion matrix were shown in Figure 4b and Figure S11, Supporting Information (details in Table S3, Supporting Information). In terms of supervised ML‐PCA method, SVM performed better than LDA and LR for differentiation between PM positive and PM negative, with AUC of 0.797, 63.0% sensitivity and 88.7% specificity at an appropriate cutoff (Figure 4b). The results of LDA and LR classifiers for ML‐PCA were shown in Figure S11, Supporting Information. Compared to supervised ML‐PCA, un‐supervised K‐PCA gained much better performance for PM detection, with AUC of 0.85, 81.5% sensitivity and 84.9% specificity at an appropriate cutoff, by using LR classifier (Figure 4b). The results of SVM and LDA classifiers for K‐PCA were shown in Figure S11, Supporting Information. As shown in Figure 4c, the PM prediction results based on ML‐PCA and K‐PCA for each patient were delineated. The directly visualized comparisons with the ground truths further demonstrated the great performance of K‐PCA based PM detection. These results suggest that our SRMC method is capable to detect PM with high sensitivity and specificity.

### 2.7 Interpretation of Feature Contribution in PM Detection by SRMC

**Figure 5:**
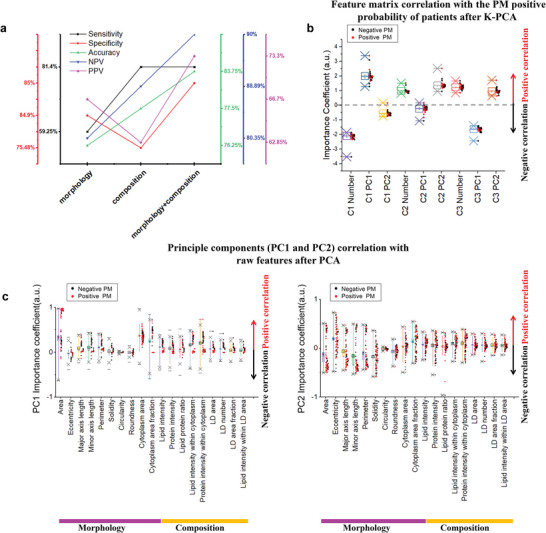
Feature contribution in PM detection by SRMC. a) Comparisons of the performance of PM detection (sensitivity, specificity, accuracy, NPV, and PPV) based on either or both of morphology and composition features. b) Importance coefficients of feature matrix to PM detection results. Feature matrix: C1 PC1: PC1 values of Cluster1, C1 Number: Cluster#1 cell number, etc. c) Importance coefficients of raw features to principal components, PC1 value (left) and PC2 value (right). The box and whisker plots represent median values (center lines), mean values (horizontal bars), minimum and maximum (outliers), 25th to 75th percentiles (box edges) and 1.5× interquartile range (whiskers), with all points plotted.

In order to interpret the result by SRMC, we analyzed importance coefficient of the single‐cell features (**Figure** **5**). The diagnostic performance of SRMC using both morphology and composition features was significantly better than that using either morphology or composition features. Specifically, the sensitivity increased from 59.25% to 81.5%, the accuracy increased from 76.25% to 83.75%, the specificity increased from 75.48% to 84.9%, negative predictive value (NPV) increased from 80.35% to 90%, and positive predictive value (PPV) increased from 62.85% to 73.3% (Figure 5a). The improved sensitivity was predominantly contributed by composition features, whereas the improved specificity was mainly contributed by morphology features. In terms of NPV and PPV, both morphology and composition features contributed.

Moreover, we analyzed the importance coefficients of feature matrix, which contributed to final PM results (Figure 5b). Importance coefficients represent the relationship between the given feature matrix and PM results, assuming that all the other matrix components remain constant (conditional dependence).^[^ ^54^ ^]^ Through analyzing importance coefficient of feature matrix by K‐PCA, the Cluster1‐number was negatively related with PM results and Cluster1‐PC1 was positively related with PM results, suggesting that clustering benefit PM detection (Figure 5b). We also quantitively analyzed the importance coefficients of raw features, suggesting the correlation between principal components and raw features. As shown in Figure 5c, principal components (PC1 and PC2) had the top three highest correlations with cellular area, cytoplasm area fraction, and protein intensity within cytoplasm. Additionally, we have analyzed the correlations between LD features (LD area, LD number, LD area fraction and lipid intensity within LD area) and cell clustering/PM diagnosis (Figure 5c). The correlations among the features of LD area, LD number and LD size were high. By adding the feature of LD size, there was no significant improvement for cell clustering/PM diagnosis. The features, including cell area, cytoplasm area, lipid intensity and protein intensity within cytoplasm etc., had positive correlations with PC1 (Figure 5c); whereas the features, including cytoplasm area, cytoplasm area fraction, lipid intensity, and protein intensity within cytoplasm, etc., had both positive and negative correlations with PC2 (Figure 5c). Therefore, our quantitative interpretation demonstrates that morphology and composition features of single exfoliated cells after clustering contribute to accurate PM detection by the SRMC method.

## 3 Discussion

In this study, we have developed the SRMC, a label‐free SRS microscopy‐based intelligent cytology method, for detection of PM in GC. Integration of three‐color SRS microscopy with deep learning segmentation model provided both morphology and composition features of single cells. Our hybrid PCA and K‐means clustering analysis further transformed these raw features to feature matrix and enabled identification of the significant marker cell population, the divergence of which was strongly related to PM. The transformed feature matrix was finally used as input features in machine learning models for PM prediction. For 80 GC patients, the SRMC achieved 81.5% sensitivity, 84.9% specificity, 83.75% accuracy, and AUC of 0.85, by using cross‐validation compared with histopathology, the gold standard of PM detection. Moreover, the average test time for each patient was within 20 min. As discussed below, our study demonstrates that the SRMC may open up a new avenue for accurate and rapid detection of PM from GC.

First, our SRMC method enhances accuracy of PM detection for GC by integrating morphology features and composition features of single exfoliated cells. Conventional peritoneal lavage cytology entirely relies on cell morphology, which reaches specificity as high as 85% but limits the sensitivity to even lower than 60% for PM diagnosis.^[^ ^16^ ^]^ This suggests that the morphology feature largely contributes to the specificity but is not enough to gain the sensitivity. Thus, the concept of well‐established SRS‐based virtual histology (SRH) method that primarily depends on morphology could not be transferred to cytology. Different from conventional cytology and SRH, our SRMC brought in composition features, which indeed significantly increased the sensitivity from 59.25% to 81.5%. Particularly, lipid protein ratio, lipid intensity within cytoplasm, and protein intensity within cytoplasm were recognized to be the most critical composition features contributing to sensitive PM detection. Additionally, we have simulated the performance of PM diagnosis by single‐color SRS (2850 cm^−1^ or 2930 cm^−1^ channel). Based on the 2850 cm^−1^ channel alone, the accuracy of the SRMC method decreased from 83.75% to 80%. Based on the 2930 cm^−1^ channel alone, the accuracy of the SRMC method decreased from 83.75% to 77.5%. The contribution of the 2850 cm^−1^ channel was greater than the 2930 cm^−1^ channel to the accuracy of SRMC method. Moreover, Liu Zhijie et al.^[^ ^48^ ^]^ developed AI‐assisted single‐shot SRS method to produce two‐color SRS images for probing both lipid and protein simultaneously. Thus, it is possible that our SRMC method could be employed on the AI‐assisted single‐shot SRS system for PM diagnosis.

Second, the performance of our SRMC method depends on accurate single cell phenotyping, which combines single cell segmentation and cell phenotyping. The single cell segmentation was conducted by using pre‐trained deep learning Stardist model to achieve high accuracy, especially for those closely touching cells that were difficult to be separated by common segmentation algorithms. The single cell phenotypes were further recognized by newly developed hybrid K‐PCA algorithm based on feature dimensionality reduction and clustering. Importantly, with specific morphology and composition features, we identified the significant marker cell cluster, the divergence of which was then used to differentiate the PM positive and negative. Similarly, a recent work demonstrated the existence of significant marker cell population at the edge between tumor and stroma for accurate prediction of tumor response to immunotherapy.^[^ ^55^ ^]^ The above evidence collectively indicates that the significant marker cells with specific molecular signatures support accurate detection of PM from GC.

Third, our SRMC method using unsupervised K‐PCA is significantly more precise than supervised ML‐PCA for PM detection. We made “cell to cell” inspections on SRMC and conventional cytology of the same specimen and classified normal/tumor cell with 93% accuracy. The 7% error of ML‐PCA based cell classification may induce bigger error for PM detection. The feature matrix transformed by K‐PCA was used to diagnosis PM with much higher accuracy. With the leave‐one‐out cross‐validation of 80 patients, LR showed the best performance for PM diagnosis among three diagnostic models (SVM, LDA and LR) (Table S4, Supporting Information). The detailed explanation was described in Note S1, Supporting Information.

Fourthly, we further investigated the mismatched results of SRMC compared with the gold standard histopathology. As shown in Table S5, Supporting Information, the results of SRMC were close to histopathology but not conventional cytology. Compared to histopathology, SRMC had 5 false negatives out of 80 patients. In order to find out the possible reason of false negative, we compared SRMC with conventional cytology on the same specimen in situ. As shown in Figure S12, Supporting Information, different from the original positive cytology result, the cytology result obtained from the same specimen analyzed by SRMC showed negative result (i.e., no tumor cells). Similarly, as shown in Figure S13, Supporting Information, different from the original negative cytology result, the cytology result obtained from the same specimen analyzed by SRMC showed positive result. These results suggest that the false negative in SRMC is possibly related with the throughput of our method and may occur in the specimen with very low percentage of tumor cells. In addition, compared to histopathology, SRMC had 8 false positives out of 80 patients, which may be due to limited features obtained from three‐color SRS imaging. The sensitivity and specificity of SRMC could be further improved by increasing throughput, for example via stimulated Raman flow cytometry,^[^ ^56^, ^57^ ^]^ and by introducing more composition features, for example via hyperspectral SRS imaging cytometry.^[^ ^58^ ^]^

Finally, our SRMC method may enable a variety of clinical practices for better management of locally advanced GC. For instance, besides rapid detection of PM during surgery, SRMC could also facilitate preoperative diagnosis and postoperative follow‐up, which can be hardly done by histopathology due to its invasiveness. As shown in Figure S14, Supporting Information, we tried to assess the changes of PM upon chemotherapy after surgery and predict prognosis. Moreover, besides PM detection, cytology itself is also a very important indicator for potential PM that may have not occurred yet. Fortunately, the SRMC is based on multi‐color SRS microscopy that has been previously established to permit virtual H&E staining.^[^ ^47^, ^48^ ^]^ Thus, the integration of SRMC with virtual staining may be desirable to give diagnostic results of both PM and cytology. Furthermore, the concept of SRMC could be extended to other clinical scenarios of cancer diagnosis using cytology, such as Pap smear for cervical cancer, urinary cytology for urothelial cancer, bronchoalveolar lavage fluid examination for lung cancer, etc. Taken together, SRMC may hold great promise for becoming the next generation of cytology in the near future.

## 4 Experimental Section

### Cell Culture

GES‐1, HMrSV5, SNU‐16 cells were cultured in RPMI 1640 (Gibco, 11875119), supplemented with 10% fetal bovine serum (Omega Scientific, FB‐21), and 0.1% penicillin/streptomycin (Gibco, 15070063). Cell cultures were incubated in an incubator at 37 °C with 5% CO_2_.

### Clinical Sample

Peritoneal lavage fluids were collected from 80 patients diagnosed with locally advanced GC in the Peking University Cancer Hospital, with 27 PM positive, 53 PM negative. and 35 CY positive, 45 CY negative. The detailed sex, age, PM positive/negative, and CY positive/negative of each patient were described in Table S3, Supporting Information. For each patient, half (100 mL) of the sample was inspected by conventional cytology by pathologists, and the other half (100 mL) was analyzed by the SRMC method. This study was approved by the Institutional Review Boards of Peking University Cancer Hospital and Beihang University. Written informed consent was obtained from all patients before the study was conducted.

### Diagnostic Laparoscopy Staging and Conventional Cytology Examination

Intraoperative laparoscopy was performed under general anesthesia. The patient was placed in a supine position. A 10‐mm disposable trocar (observing hole) was inserted into the sub‐umbilicus, and a 30° telescope was used. Another 10‐mm trocar and a 5‐mm trocar were inserted into the right and left upper quadrants, respectively. Prior to any manipulation, 250 mL of warm normal saline was infused into the subphrenic space, subhepatic space, momentum, bilateral paracolic sulci, and the pouch of Douglas. Care was taken to avoid direct contact of the irrigation with the primary tumor. At least 200 mL of fluid was aspirated from the subphrenic space, subhepatic space and pouch of Douglas. The fluid was immediately sent for the SRMC and cytological examination. For conventional cytology, the exfoliated cells were stained with hematoxylin and eosin (H&E). Two professional pathologists examined the H&E slides independently. Patients with negative cytology results were labeled as CY−, and patients with positive cytology results were labeled as CY+. Subsequently, a systematic inspection of the abdominal cavity was performed clockwise from the right quadrant. Any suspicious lesion was biopsied and sent for histopathologic examination. Patients with negative histopathologic results were diagnosed as PM negative (PM−), and patients with positive histopathologic results were diagnosed as PM positive (PM+).

### Preparation of the Exfoliated Cell for SRS Imaging

The ascites samples were first centrifuged at 2000 rpm for 3 min. Then, the concentrated samples were treated by red blood cell lysate for 5 min and rinsed with phosphate buffered saline. The sample (≈10 µL) was dropped on the glass slide and evenly smeared in an area of about 1 cm^2^. The slide was used for SRS imaging after air‐drying and stored at −80 °C for future use if needed.

### Three‐Color SRS Microscopy

The authors’ SRS microscope employed a dual output picosecond pulse laser (picoEmeraldTM S, Applied Physics and Electronics) with a repetition rate at 80 MHz and 2 ps width. The laser has an integrated output for both the pump beam with tunable wavelength from 700 nm to 960 nm and the Stokes beam with fixed wavelength at 1031 nm, which are overlapped in space and time. When performing SRS, the Stokes beam was modulated at ≈20 MHz by an electronic optic modulator. The collinear pump and Stokes beams were coupled to a 2D scanning galvanometer (GVS012‐2D, Thorlabs) and then imported into an inverted microscope (IX73, Olympus). A 60× water immersion objective lens (LUMPlanFL N, 1.0 numerical aperture, Olympus) focused the lasers into the sample. The photons were collected by a 60× water condenser (LUMPlanFL N, 1.0 numerical aperture, Olympus). The pump beam was selected by a short‐pass filter (ET980SP, Chroma), and was detected by a photodiode (S3994‐01, Hamamatsu, Japan) equipped with a resonant circuit that selectively amplifies the signal at the optical modulation frequency. The stimulated Raman loss signal was then extracted by a digital lock‐in amplifier (HF2LI, Zurich Instrument, Zurich, Switzerland). The output voltage from the lock‐in amplifier, which represents the SRS signal, was sampled by a DAQ card (PCI‐ 6363, National Instruments, Austin, TX). A LabVIEW platform synchronized scanning of wavelength with 2D multivariable acquisition of XY images. SRS imaging was performed by tuning 796.8, 791.8, and 789.5 nm to get the Raman band of 2850, 2930, and 2965 cm^−1^, which correspond to the C—H stretching region of lipid, protein, and DNA, respectively. The excitation power at the sample was ≈20 mW for pump and ≈90 mW for Stokes for all cell samples. Each SRS image contained 400 × 400 pixels. The pixel dwell time was 10 µs. The field of view of single SRS images was 100 × 100 µm.

### Image Processing and Analysis

For SRS image processing, home‐built Python programs were used to segment single cell with masks, extract features, and classify single cell with labelled masks. The whole process was described in Table S6, Supporting Information.

The single cell segmentation ran with the Python library stardist2D 0.7.1 under tensorflow 1.7. The feature extraction ran with the Python library skimage 0.18.0. The cell phenotyping and dimensional reduction using K‐PCA and ML‐PCA ran with the Python library sklearn 0.21.2. PCA as a linear dimensionality reduction uses singular value decomposition (SVD) of the features data to project it to a lower dimensional space. The input feature data was centered but not scaled for each feature before applying the SVD. The K‐means algorithm clustered input features data by separating cells in *n* groups of equal variances, through minimizing a criterion known as sum of squares within cluster.

After cell phenotyping, labelled masks were created and saved with virtual pseudo colors. The saved ROI files could be opened by the ImageJ. The significant feature components (cellular area, perimeter, cytoplasm area, lipid intensity, protein intensity, and lipid protein ratio etc.) of three clusters were saved in csv files. Imported with the significant components, the supervised classifiers (SVM, LR, and LDA) all ran with the Python library sklearn 0.21.2.

The positive probability of PM, confusion matrix and ROC curve for each of the 80 patients were plotted and saved. The whole image processing and analysis (Table S6, Supporting Information) for each patient with 500 cells took less than 1 min on a customized computer equipped with a single graphics card (EVGA GeForce GTX 2080Ti FTW3) and an Intel i9‐7920x CPU.

### Large‐Scale SRS Imaging

A home‐built code was programmed for large‐scale SRS imaging including automatic scanning, automatic stitching, and automatic calibration. Automatic scanning was programmed by home‐built LabVIEW software. 4 × 10 images were acquired with 20% overlay to create an FOV of 400 × 1000 µm^2^. After image acquisition, the stitching algorithm programmed by MATLAB automatedly calibrated and smoothed the edges between images. The shifts among three‐color SRS image channels were corrected. The schematic and typical results were described in the Figure S7, Supporting Information.

### Extraction of Features from Single Cells

19 features were totally extracted, including morphology and composition, of single exfoliated cells, as shown in Figure S2, Supporting Information. The morphology features included area, eccentricity, major axis length, minor axis length, perimeter, solidity, circularity, roundness, cytoplasm area fraction, and cytoplasm area. Major and minor axis length were the primary and secondary axis of the best fitting ellipse with the selection cell contour. The solidity was convex area of the convex hull that encloses single cell divided by cellular area. The eccentricity was minor axis length divided by major axis length. The circularity and roundness were calculated by the following equations, respectively.

(1) $$\mathrm{Circularity}=\frac{4\pi \times \mathrm{area}}{\mathrm{perimeter}}$$

(2) $$\mathrm{Roundness}=\frac{4\times \mathrm{area}}{\pi \times \mathrm{major}\;\mathrm{axis}\;{\mathrm{length}}^{2}}$$

The composition features included lipid intensity, protein intensity, lipid protein ratio, lipid intensity within cytoplasm, the protein intensity within cytoplasm, lipid droplet (LD) area, LD number, LD area fraction, and lipid intensity within LD area. The lipid and protein intensity of each image were calculated from the intensity of SRS image at 2850 cm^−1^ and 2930 cm^−1^ by the following formula.

(3) $$\left(\begin{array}{c} C_{L}\\ C_{P} \end{array}\right)=\left(\begin{array}{c} 1.28583\;\;-0.32401\\ -0.13406\;\;1.28583 \end{array}\right)\left(\begin{array}{c} S_{L}\\ S_{P} \end{array}\right)$$

*S*_L_ is the signal intensity of SRS image at 2850 cm^−1^, *S*_P_ is the signal intensity of SRS image at 2930 cm^−1^, *C*_L_ is the lipid intensity, and *C*_P_ is the protein intensity of each image. The constants were measured from pure BSA and oil by SRS imaging at 2930 cm^−1^ and 2850 cm^−1^.

As shown in Figure S2, Supporting Information, the 8 morphology features such as area, eccentricity, major axis length, minor axis length, perimeter, solidity, circularity and roundness were extracted from cellular masks by using skimage.measure function in Python script as same as the “Measure” module at ImageJ after single cell segmentation.

Then, the lipid intensity, protein intensity, and lipid protein ratio of single cells were also extracted using skimage.measure function based on *C*_L_, *C*_P_, and cellular masks. Then, nucleus segmentation masks were extracted from DNA channel by adaptive thresholding. By using skimage.measure function, cytoplasm area were calculated by cytoplasm masks, which are cell masks subtracted by nucleus masks. Cytoplasm area fraction was cytoplasm area divided by cell area. Then another two composition features (lipid intensity within cytoplasm, and protein intensity within cytoplasm) were calculated by *C*_L_, *C*_P_, and cytoplasm masks.

Finally, masks of LD area were obtained from 2850 cm^−1^ by Python library cv2.threshold, as same as the “Threshold” module at ImageJ. LD in single cells could be segmented due to their higher local signal intensities compared to surrounding cellular compartments. By using numpy.percentile function in Python script, intensity threshold value of each image could be obtained from the overall intensity histogram. Then based on LD masks, the LD number and LD area were extracted. LD area fraction was LD area divided by cell area. Lipid intensity within LD area was calculated by *C*_L_ and LD mask. The segmented LDs areas were highly consistent with visual judgment as shown in Figure S2, Supporting Information.

### Quantitative Evaluation of Single Cell Segmentation

Dice parameter, relative mean square error (RMSE) between ground truth and automate cell segmentation were used to quantitatively assess the performance of cell segmentation. Dice parameter represents the overlap of segmentation area by stardist 2D and visual judgment by the formula.

(4) $$\mathrm{Dice}=\frac{2\left|\left.\mathrm{stardist}\cap \mathrm{visual}\right|\right.}{\left|\left.\mathrm{stardist}\right|\right.+\left|\left.\mathrm{visual}\right|\right.}$$

RMSE represents relative mean square error of cell counting between stardist 2D and visual judgment in each image.

(5) $$\mathrm{RMSE}=\frac{1}{N}\sum_{i=1}^{N}\sqrt{{\left(n_{i}^{\mathrm{stardist}}-n_{i}^{\mathrm{visual}}\right)}^{2}}$$

*n_i_* ^stardist^ means cell counting number by stardist model and *n_i_* ^viusal^ means cell number from visual judgement in each image *i*.

### Statistical Analysis

The box and whisker plots with Origin 2017 represent median values (center lines), mean values (horizontal bars), minimum and maximum (outliers), 25th to 75th percentiles (box edges), and 1.5× interquartile range (whiskers), with all points plotted. Student's *t*‐test was used for comparisons between groups. The formula for the two‐sample *t*‐test (Student's *t*‐test) is shown below.

(6) $$t=\frac{x_{1}-x_{2}}{\sqrt{s_{2}\left(\frac{1}{n_{1}}+\frac{1}{n_{2}}\right)}}$$

In this formula, *t* is the *t* value, *x*_1_ and *x*_2_ are the means of the two groups, *s*_2_ is the standard error of the two groups, and *n*_1_ and *n*_2_ are the number in each of two groups. Here, the *t*‐value was to quantify the difference between two group means. A larger *t*‐value showed that the difference between the group means was greater than the standard error, indicating a more significant difference between two groups. The *p*‐value was the conditional probability of obtaining at least as *t*‐value from data distribution of two groups, and *p*‐value < 0.05 was considered statistically significant. *t*‐value and *p*‐value were performed by Origin 2017 using Student's *t*‐test.

The positive and negative classification for PM was evaluated with ROC curve analysis. The area under the ROC curve (AUC) ranging from 0 to 1 evaluated the ability of a model to accurately distinguish two categories. A threshold of Cluster1‐PC1 and Cluster1‐PC2 respectively in Figure S5, Supporting Information, for determining PM NEG/POS was obtained by the ROC curves to get the best diagnostic sensitivity and specificity. The ROC analysis was performed by Origin 2017.

## Conflict of Interest

The authors declare no conflict of interest.

## Supporting information

Supporting Information

## Data Availability

The data that support the findings of this study are available from the corresponding author upon reasonable request.

## References

[1] H. Sung , J. Ferlay , R. L. Siegel , M. Laversanne , I. Soerjomataram , A. Jemal , F. Bray , CA Cancer J. Clin. 2021, 71, 209. 33538338 10.3322/caac.21660

[2] H. H. Hartgrink , E. P. Jansen , N. C. Van Grieken , C. J. Van De Velde , Lancet 2009, 374, 477. 19625077 10.1016/S0140-6736(09)60617-6 PMC4613761

[3] E. V. Cutsem , X. Sagaert , B. Topal , K. Haustermans , H. Prenen , Lancet Oncol. 2016, 388, 2654. 10.1016/S0140-6736(16)30354-3 27156933

[4] S. D. Lee , K. W. Ryu , B. W. Eom , J. H. Lee , M. C. Kook , Y.‐W. Kim , Br. J. Surg. 2012, 99, 397. 22101572 10.1002/bjs.7812

[5] E. Higaki , S. Yanagi , N. Gotohda , T. Kinoshita , T. Kuwata , M. Nagino , A. Ochiai , S. Fujii , Cancer Sci. 2017, 108, 978. 28256061 10.1111/cas.13219 PMC5448654

[6] Y. Yonemura , E. Bandou , T. Kawamura , Y. Endou , T. Sasaki , Eur. J. Surg. Oncol. 2006, 32, 602. 16617004 10.1016/j.ejso.2006.03.003

[7] S. Burbidge , K. Mahady , K. Naik , Clin. Radiol. 2013, 68, 251. 22985749 10.1016/j.crad.2012.07.015

[8] S. J. Kim , H.‐H. Kim , Y. H. Kim , S. H. Hwang , H. S. Lee , D. J. Park , S. Y. Kim , K. H. Lee , Radiology 2009, 253, 407. 19789243 10.1148/radiol.2532082272

[9] C. J. Allen , T. E. Newhook , T. J. Vreeland , P. Das , B. D. Minsky , M. Blum , S. Song , J. Ajani , N. Ikoma , P. F. Mansfield , S. Roy‐Chowdhuri , B. D. Badgwell , J. Surg. Oncol. 2019, 120, 1350. 31612494 10.1002/jso.25729

[10] Y. Zheng , D. Xu , Z. Bu , Chin. J. Cancer Res. 2016, 28, 144. 27041936 10.3978/j.issn.1000-9604.2016.02.10 PMC4779760

[11] Japanese Gastric Cancer Association , Gastric Cancer 2021, 24, 21. 10.1007/s101209800016 11957040

[12] J. A. Ajani , T. A. D'amico , D. J. Bentrem , J. Chao , D. Cooke , C. Corvera , P. Das , P C. Enzinger , T. Enzler , P. Fanta , F. Farjah , H. Gerdes , M K. Gibson , S. Hochwald , W L. Hofstetter , D H. Ilson , R N. Keswani , S. Kim , L R. Kleinberg , S J. Klempner , J. Lacy , Q P. Ly , K A. Matkowskyj , M. Mcnamara , M F. Mulcahy , D. Outlaw , H. Park , K A. Perry , J. Pimiento , G A. Poultsides , et al., J. Natl. Compr. Cancer Network 2022, 20, 167. 10.6004/jnccn.2022.0008 35130500

[13] Z. Li , Z. Li , L. Zhang , Q. Liu , Z. Wang , Z. Zhang , G. Xiao , W. Fu , X. Wang , Y. Ye , J. Yu , F. Li , L. Chen , S. Wang , J. Ji , BMC Cancer 2018, 18, 63. 29321007 10.1186/s12885-017-3791-6 PMC5764004

[14] J. Nath , K. Moorthy , P. Taniere , M. Hallissey , D. Alderson , Br. J. Surg. 2008, 95, 721. 18412292 10.1002/bjs.6107

[15] E. Cotte , P. Peyrat , E. Piaton , F. Chapuis , M. Rivoire , O. Glehen , C. Arvieux , J.‐Y. Mabrut , J. Chipponi , F.‐N. Gilly , Eur. J. Surg. Oncol. 2013, 39, 707. 23601984 10.1016/j.ejso.2013.03.021

[16] D. Hoskovec , J. Varga , P. Dytrych , E. Konecna , J. Matek , Arch. Med. Sci. 2017, 13, 612. 28507577 10.5114/aoms.2016.64044 PMC5420630

[17] E. Bando , Y. Yonemura , K. Taniguchi , T. Yasui , S. Fushida , T. Fujimura , G.‐I. Nishimura , K. Miwa , Am. J. Surg. 1999, 178, 256. 10527450 10.1016/s0002-9610(99)00162-2

[18] S. ‘. Abe , H. Yoshimura , H. Tabara , M. Tachibana , N. Monden , T. Nakamura , S. Nagaoka , J. Surg. Oncol. 1995, 59, 226. 7630168 10.1002/jso.2930590405

[19] S. Kagawa , K. Shigeyasu , M. Ishida , M. Watanabe , H. Tazawa , T. Nagasaka , Y. Shirakawa , T. Fujiwara , World J. Gastroenterol. 2014, 20, 17796. 25548478 10.3748/wjg.v20.i47.17796 PMC4273130

[20] V. Sood , C. Collins , S. Harrington , A. Hahn , A. Ata , A. Mapara‐Shah , W. Wang , W. Dunnican , Surg. Endosc. 2012, 26, 189. 21847695 10.1007/s00464-011-1853-4

[21] Y. Zhang , J. Zhao , H. Yu , P. Li , W. Liang , Z. Liu , G.‐B. Lee , L. Liu , W. J. Li , Z. Wang , Sci. Adv. 2020, 6, eaba9628. 32821829 10.1126/sciadv.aba9628 PMC7406364

[22] J. Kitayama , S. Emoto , H. Yamaguchi , H. Ishigami , T. Kamei , H. Yamashita , Y. Seto , K. Matsuzaki , T. Watanabe , Cytometry B Clin. Cytom. 2014, 86, n/a. 10.1002/cyto.b.21126 24115348

[23] J. Wong , K. J. Kelly , A. Mittra , M. Gonen , P. Allen , Y. Fong , D. Coit , J. Gastrointest. Surg. 2012, 16, 889. 22362071 10.1007/s11605-012-1845-2

[24] A. Wang , Z. Li , Q. Wang , Y. Bai , X. Ji , T. Fu , Ke Ji , Y. Xue , T. Han , X. Wu , Ji Zhang , Y. Yang , G. Xu , Z. Bu , J. Ji , Chin. J. Cancer Res. 2019, 31, 945. 31949396 10.21147/j.issn.1000-9604.2019.06.10 PMC6955163

[25] H.‐D. Chae , In‐H Kim , Scand. J. Gastroenterol. 2016, 51, 956. 27162096 10.3109/00365521.2016.1172339

[26] J.‐Y. Wang , S.‐R. Lin , C.‐Y. Lu , C.‐C. Chen , D.‐C. Wu , C.‐Y. Chai , F.‐M. Chen , J.‐S. Hsieh , T.‐J. Huang , Cancer Lett. 2005, 223, 129. 15890245 10.1016/j.canlet.2004.09.031

[27] Y. Kodera , H. Nakanishi , S. Ito , Y. Yamamura , Y. Kanemitsu , Y. Shimizu , T. Hirai , K. Yasui , T. Kato , M. Tatematsu , Ann. Surg. 2002, 235, 499. 11923605 10.1097/00000658-200204000-00007 PMC1422464

[28] K. Mori , T. Suzuki , H. Uozaki , H. Nakanishi , T. Ueda , Y. Matsuno , Y. Kodera , H. Sakamoto , N. Yamamoto , M. Sasako , M. Kaminishi , H. Sasaki , Ann. Surg. Oncol. 2007, 14, 1694. 17294072 10.1245/s10434-006-9321-4

[29] N. N. Pavlova , J. Zhu , C. B. Thompson , Cell Metab. 2022, 34, 355. 35123658 10.1016/j.cmet.2022.01.007 PMC8891094

[30] L. A. Broadfield , A. A. Pane , A. Talebi , J. V. Swinnen , S.‐M. Fendt , Dev. Cell 2021, 56, 1363. 33945792 10.1016/j.devcel.2021.04.013

[31] T. Ueo , H. Yonemasu , N. Yada , S. Yano , T. Ishida , M. Urabe , K. Takahashi , H. Nagamatsu , R. Narita , K. Yao , T. Daa , S. Yokoyama , Dig. Endosc. 2013, 25, 147. 23368762 10.1111/j.1443-1661.2012.01364.x

[32] M.‐Y. Cui , X. Yi , D.‐X. Zhu , J. Wu , Front. Oncol. 2022, 12, 916661. 35785165 10.3389/fonc.2022.916661 PMC9240397

[33] G. Bergers , S.‐M. Fendt , Nat. Rev. Cancer 2021, 21, 162. 33462499 10.1038/s41568-020-00320-2 PMC8733955

[34] X. Luo , C. Cheng , Z. Tan , N. Li , M. Tang , L. Yang , Ya Cao , Mol. Cancer 2017, 16, 76. 28399876 10.1186/s12943-017-0646-3 PMC5387196

[35] G. Pan , Y. Ma , J. Suo , W. Li , Y. Zhang , S. Qin , Y. Jiao , S. Zhang , S. Li , Y. Kong , Yu Du , S. Gao , D. Wang , Onco Targets Ther. 2020, 13, 7199. 32801750 10.2147/OTT.S245663 PMC7394602

[36] D. Traynor , I. Behl , D. O'dea , F. Bonnier , S. Nicholson , F. O'connell , A. Maguire , S. Flint , S. Galvin , C. M. Healy , C. M. Martin , J. J. O'leary , A. Malkin , H. J. Byrne , F. M. Lyng , Nat. Protoc. 2021, 16, 3716. 34117476 10.1038/s41596-021-00559-5

[37] H. K. Yosef , S. D. Krauß , T. Lechtonen , H. Jütte , A. Tannapfel , H. U. Käfferlein , T. Brüning , F. Roghmann , J. Noldus , A. Mosig , S. F. El‐Mashtoly , K. Gerwert , Anal. Chem. 2017, 89, 6893. 28541036 10.1021/acs.analchem.7b01403

[38] H. J. Byrne , I. Behl , G. Calado , O. Ibrahim , M. Toner , S. Galvin , C. M. Healy , S. Flint , F. M. Lyng , Spectrochim. Acta, Part A 2021, 252, 119470. 10.1016/j.saa.2021.119470 33503511

[39] Y. Qi , G. Zhang , L. Yang , B. Liu , H. Zeng , Qi Xue , D. Liu , Q. Zheng , Y. Liu , Anal. Chem. 2022, 94, 6491. 35271250 10.1021/acs.analchem.1c05098

[40] J.‐X. Cheng , X. S. Xie , Science 2015, 350, aaa8870. 26612955 10.1126/science.aaa8870

[41] Stimulated Raman Scattering Microscopy (Eds: J.‐X. Cheng , W. Min , Y. Ozeki , D. Polli ), Elsevier, Amsterdam 2022.

[42] Y. Shen , F. Hu , W. Min , Annu. Rev. Biophys. 2019, 48, 347. 30892920 10.1146/annurev-biophys-052118-115500

[43] S. Yue , in Stimulated Raman Scattering Microscopy (Eds: J.‐X. Cheng , W. Min , Y. Ozeki , D. Polli ), Elsevier, Amsterdam 2022, pp. 463–473.

[44] A. Moskalik , Y. Dastagirzada , D. Orringer , in Stimulated Raman Scattering Microscopy (Eds: J.‐X. Cheng , W. Min , Y. Ozeki , D. Polli ), Elsevier, Amsterdam 2022, pp. 541–549.

[45] M. Ji , D. A. Orringer , C. W. Freudiger , S. Ramkissoon , X. Liu , D. Lau , A. J. Golby , I. Norton , M. Hayashi , N. Y. R. Agar , G. S. Young , C. Spino , S. Santagata , S. Camelo‐Piragua , K. L. Ligon , O. Sagher , X. S Xie , Sci. Transl. Med. 2013, 5, 201ra119. 10.1126/scitranslmed.3005954 PMC3806096 24005159

[46] M. Ji , S. Lewis , S. Camelo‐Piragua , S. H. Ramkissoon , M. Snuderl , S. Venneti , A. Fisher‐Hubbard , M. Garrard , D. Fu , A. C. Wang , J. A. Heth , C. O. Maher , N. Sanai , T. D. Johnson , C. W. Freudiger , O. Sagher , X. S. Xie , D. A. Orringer , Sci. Transl. Med. 2015, 7, 309ra163. 10.1126/scitranslmed.aab0195 PMC4900155 26468325

[47] T. C. Hollon , B. Pandian , A. R. Adapa , E. Urias , A. V. Save , S. S. S. Khalsa , D. G. Eichberg , R. S. D'amico , Z. U. Farooq , S. Lewis , P. D. Petridis , T. Marie , A. H. Shah , H. J. L. Garton , C. O. Maher , J. A. Heth , E. L. Mckean , S. E. Sullivan , S. L. Hervey‐Jumper , P. G. Patil , B. G Thompson , O. Sagher , G. M. Mckhann , R. J. Komotar , M. E. Ivan , M. Snuderl , M. L. Otten , T. D. Johnson , M. B. Sisti , J. N. Bruce , et al., Nat. Med. 2020, 26, 52. 31907460 10.1038/s41591-019-0715-9 PMC6960329

[48] Z. Liu , W. Su , J. Ao , M. Wang , Q. Jiang , J. He , H. Gao , S. Lei , J. Nie , X. Yan , X. Guo , P. Zhou , H. Hu , M. Ji , Nat. Commun. 2022, 13, 4050. 35831299 10.1038/s41467-022-31339-8 PMC9279377

[49] L. Zhang , Y. Wu , B. Zheng , L. Su , Y. Chen , S. Ma , Q. Hu , X. Zou , L. Yao , Y. Yang , L. Chen , Y. Mao , Y. Chen , M. Ji , Theranostics 2019, 9, 2541. 31131052 10.7150/thno.32655 PMC6526002

[50] D. A. Orringer , et al., Nat. Biomed. Eng. 2017, 1, 0027. 31015705 10.1038/s41551-017-0172-z

[51] R. C. Hoesli , D. A. Orringer , J. B. Mchugh , M. E. Spector , Otolaryngol. Head Neck Surg. 2017, 157, 448. 28397572 10.1177/0194599817700388 PMC5646167

[52] B. Sarri , F. Poizat , S. Heuke , J. Wojak , F. Franchi , F. Caillol , M. Giovannini , H. Rigneault , Biomed. Opt. Express 2019, 10, 5378. 31646052 10.1364/BOE.10.005378 PMC6788596

[53] Y. Yu , Ke Lin , X. Zhou , H. Wang , S. Liu , X. Ma , J. Phys. Chem. C 2007, 111, 8971.

[54] Inria Learning Lab . 2022. Feature importance – Scikit‐learn course. https://inria.github.io/scikit‐learn‐mooc/python_scripts/dev_features_importance.html.

[55] S. Berry , N. A. Giraldo , B. F. Green , T. R. Cottrell , J. E. Stein , E. L. Engle , H. Xu , A. Ogurtsova , C. Roberts , D. Wang , P. Nguyen , Q. Zhu , S. Soto‐Diaz , J. Loyola , I. B. Sander , P. F. Wong , S. Jessel , J. Doyle , D. Signer , R. Wilton , J. S. Roskes , M. Eminizer , S. Park , J. C. Sunshine , E. M. Jaffee , A. Baras , A. M. De Marzo , S. L. Topalian , H. Kluger , L. Cope , et al., Science 2021, 372, eaba2609. 34112666 10.1126/science.aba2609 PMC8709533

[56] C. Zhang , K.‐C. Huang , B. Rajwa , J. Li , S. Yang , H. Lin , C.‐S. Liao , G. Eakins , S. Kuang , V. Patsekin , J. P Robinson , J.‐X Cheng , Optica 2017, 4, 103. 10.1364/optica.4.000103 PMC11375991 39238893

[57] Y. Suzuki , K. Kobayashi , Y. Wakisaka , D. Deng , S. Tanaka , C.‐J. Huang , C. Lei , C.‐W. Sun , H. Liu , Y. Fujiwaki , S. Lee , A. Isozaki , Y. Kasai , T. Hayakawa , S. Sakuma , F. Arai , K. Koizumi , H. Tezuka , M. Inaba , K. Hiraki , T. Ito , M. Hase , S. Matsusaka , K. Shiba , K. Suga , M. Nishikawa , M. Jona , Y. Yatomi , Y. Yalikun , Y. Tanaka , et al., Proc. Natl. Acad. Sci. U. S. A. 2019, 116, 15842. 31324741 10.1073/pnas.1902322116 PMC6690022

[58] K.‐C. Huang , J. Li , C. Zhang , Y. Tan , J.‐X Cheng , iScience 2020, 23, 100953. 32179477 10.1016/j.isci.2020.100953 PMC7078382

