# Supplementary material for: Accurate and Rapid Detection of Peritoneal Metastasis from Gastric Cancer by AI‐Assisted Stimulated Raman Molecular Cytology
Source: Adv Sci (Weinh). 2023 Apr 28;10(21):2300961. doi: 10.1002/advs.202300961 (PMC10375130; doi:10.1002/advs.202300961)
Supplement: Supplementary file 1 — Supporting Information [file ADVS-10-2300961-s001.pdf]

ADVANCED SCIENCE
Open Access

Supporting Information

for *Adv. Sci.*, DOI 10.1002/advs.202300961

Accurate and Rapid Detection of Peritoneal Metastasis from Gastric Cancer by AI-Assisted Stimulated Raman Molecular Cytology

Xun Chen, Zhouqiao Wu, Yexuan He, Zhe Hao, Qi Wang, Keji Zhou, Wanhui Zhou, Pu Wang, Fei Shan, Zhongwu Li, Jiafu Ji, Yubo Fan, Ziyu Li* and Shuhua Yue**

Supplementary materials:

Accurate and Rapid Detection of Peritoneal Metastasis from Gastric Cancer by AI-assisted Stimulated Raman Molecular Cytology

Xun Chen[1,3,&], Zhouqiao Wu[2,&,] Yexuan He[1], Zhe Hao[1], Qi Wang[2], Keji Zhou[1], Wanhui Zhou[1], Pu Wang[1], Fei Shan[2], Zhongwu Li[4], Jiafu Ji[2], Yubo Fan[1,3]*, Ziyu Li[2]* and Shuhua Yue[1]*

[1]Key Laboratory of Biomechanics and Mechanobiology (Beihang University), Ministry of Education, Institute of Medical Photonics, Beijing Advanced Innovation Center for Biomedical Engineering, School of Biological Science and Medical Engineering, Beihang University, Beijing 100083, China
[2]Gastrointestinal Cancer Center, Key Laboratory of Carcinogenesis and Translational Research (Ministry of Education), Peking University Cancer Hospital & Institute, Beijing 100142, China
[3]School of Engineering Medicine, Beihang University, Beijing 100083, China
[4] Key Laboratory of Carcinogenesis and Translational Research (Ministry of Education), Department of Pathology, Peking University Cancer Hospital & Institute, Beijing 100142, China
[&]These authors contributed equally.
*Corresponding authors: yue_shuhua@buaa.edu.cn; ziyu_li@hsc.pku.edu.cn; yubofan@buaa.edu.cn

Supplementary Materials

Supplementary Note 1: Cell phenotyping with SRMC and H&E cytology of the same sample

Supplementary Fig. S1. Performance of single cell segmentation by stardist model.

Supplementary Fig. S2. Workflow of feature extraction based on masks.

Supplementary Fig. S3. Results of SRMC of gastric cell lines with single cell segmentation and classification.

Supplementary Fig. S4. Representative SRS imaging of GC exfoliated cells and comparisons of 19 features between positive and negative PM.

Supplementary Fig. S5. Hot map of raw feature and feature matrix transformed by K-PCA, and ROC analysis of feature matrix.

Supplementary Fig. S6. Representative images and quantitative analysis of exfoliated cell phenotyping.

Supplementary Fig. S7. Image stitching of three-color SRS imaging of exfoliated cells.

Supplementary Fig. S8. In situ SRS&HE imaging of exfoliated cells.

Supplementary Fig. S9. Typical tumor cell and normal cell detection by ML-PCA.

Supplementary Fig. S10. Results of feature profiles by K-PCA and ML-PCA.

Supplementary Fig. S11. Results of PM detection by classifiers (SVM, LDA, LR etc.).

Supplementary Fig. S12. Typical SRS&HE in-situ images for SRMC negative detection.

Supplementary Fig. S13. Typical SRS&HE in-situ images for SRMC positive detection.

Supplementary Fig. S14. PM positive probability before and after chemotherapy for patients diagnosed with PM positive (n = 3) and PM negative (n=2).

Supplementary Table S1. Typical raw features of 80 patients.

Supplementary Table S2. Values of Cluster1-PC1, Cluster1-PC2 and Cluster2-PC1, Cluster2-PC2 of 80 patients.

Supplementary Table S3. Diagnostic results for each patient, including conventional cytology, histopathology, and SRMC (based on SVM, LDA, or LR models) predicted probability of PM.

Supplementary Table S4. Performance comparisons between K-PCA and ML-PCA methods.

Supplementary Table S5. Comparisons between our SRMC results and conventional cytology, histopathology, respectively.

Supplementary Table S6. Image acquisition and processing workflow by task, software, and description.

Supplementary Note1: Cell phenotyping with SRMC and H&E cytology of the same sample

We compared the performance of cell phenotypes algorithms using in situ SRS&HE cytology. We firstly imaged the exfoliated cells using stimulated Raman molecular cytology (SRMC). Then we labeled the samples with H&E dye and imaged in situ cells of same slices. Using SRMC method, we stitched 4*10 FOVs to create a large scope comparable with traditional H&E cytology with a customized MATLAB program. The imaging results of the label-free exfoliated cells were compared with cytological images in situ with hematoxylin and eosin (H&E) staining by "cell to cell" (**Supplementary Fig. S8**). Based on the single cell labelling from pathologists, we built a machine learning based model (LDA and SVM) to classify normal/tumor cells. The training and test dataset (8:2) include total 12620 cells (1158 normal cells; 11462 tumor cells). The best accuracy of single cell classification was could be 93.8% after our model optimization.

We also studied the cell phenotype algorithms for PM detection using K-PCA and ML-PCA (**Supplementary Fig. S9 and Fig. 3**). Using K-PCA algorithm, we could filter out significant marker cells from bundle of normal/tumor cells (**Supplementary Fig. S9 and Fig. 3**). For PM positive with lots of tumor cells, the composition features (LD number) and shape features (cellular area and cytoplasm area fraction) of significant marker cells (*Cluster 1*) were significantly different than other tumor cells (*Cluster 2&3*) (**Supplementary Fig S10**). For PM positive with few tumor cells, the feature divergence between clusters becomes weaker. Meanwhile, cellular area, lipid intensity, cytoplasm area fraction and LD number after ML-PCA become more significantly different than K-PCA. Additionally, the lipid intensity, LD number of tumor cells are higher than normal cells, which are consistent with previous studies[1]. For PM negative without tumor cells, cellular area, LD number, and cytoplasm area fraction of significant maker cells (*Cluster 1*) were significantly different than other normal cells (*Cluster 2&3*) (**Supplementary Fig S10**).

We also compared the features of shape, composition especially LDs, and the raw features were mixed between positive/negative PM before K-PCA. After K-PCA based dimensional reduction, the positive/negative PM were obviously separated with *Cluster1*-number and *Clutser1*-PC1 (**Supplementary Fig. S5**). The final PM detection results from cell phenotype were shown in **Fig. 4** and **Supplementary Fig. S11**. Incorporating with LDA, SVM and LR classifiers, the unsupervised K-PCA for PM detection was better than supervised ML-PCA with leave-one-out cross-validation. Specially, the sensitivity of unsupervised K-PCA method performs better (**Supplementary Fig. S11** and **table S4**). The final PM detection accuracy of K-PCA with LR, SVM and LDA were respectively 83.75%, 78.75% and 77.5%%; ML-PCA were 80%, 72.5% and 76.25% respectively as shown in **Fig. 4** and **Supplementary Fig. S11**.

By using SRS&HE in situ method, for some false positive patients, and the mismatch patient between primary CY method and SRMC method also were discussed in **Supplementary Fig. S12** and **Fig. S13**. In **Supplementary table S5**, it demonstrated that false positive (8/80) of SRMC method or false negative (5/80) of SRMC method, comparing with PM results. In situ CY&SRMC result (2/80) confirms there is no tumor cell of our detection cells when SRMC mismatched with PM to get false negative. False negative also may be induced by the bias of sample random collection of ascites. If the high throughput SRMC cell detection enables in future, we may improve this condition. Moreover, the PM positive rate of gastric patients decreased significantly after chemotherapy (**Supplementary Fig. S14**), and positive probability of 3 PM+ patient decreased from 85% to 38%; 2 PM- patient decreased from 38% to 27%. This indicates that our SRMC method may effectively assess chemotherapy prognosis.

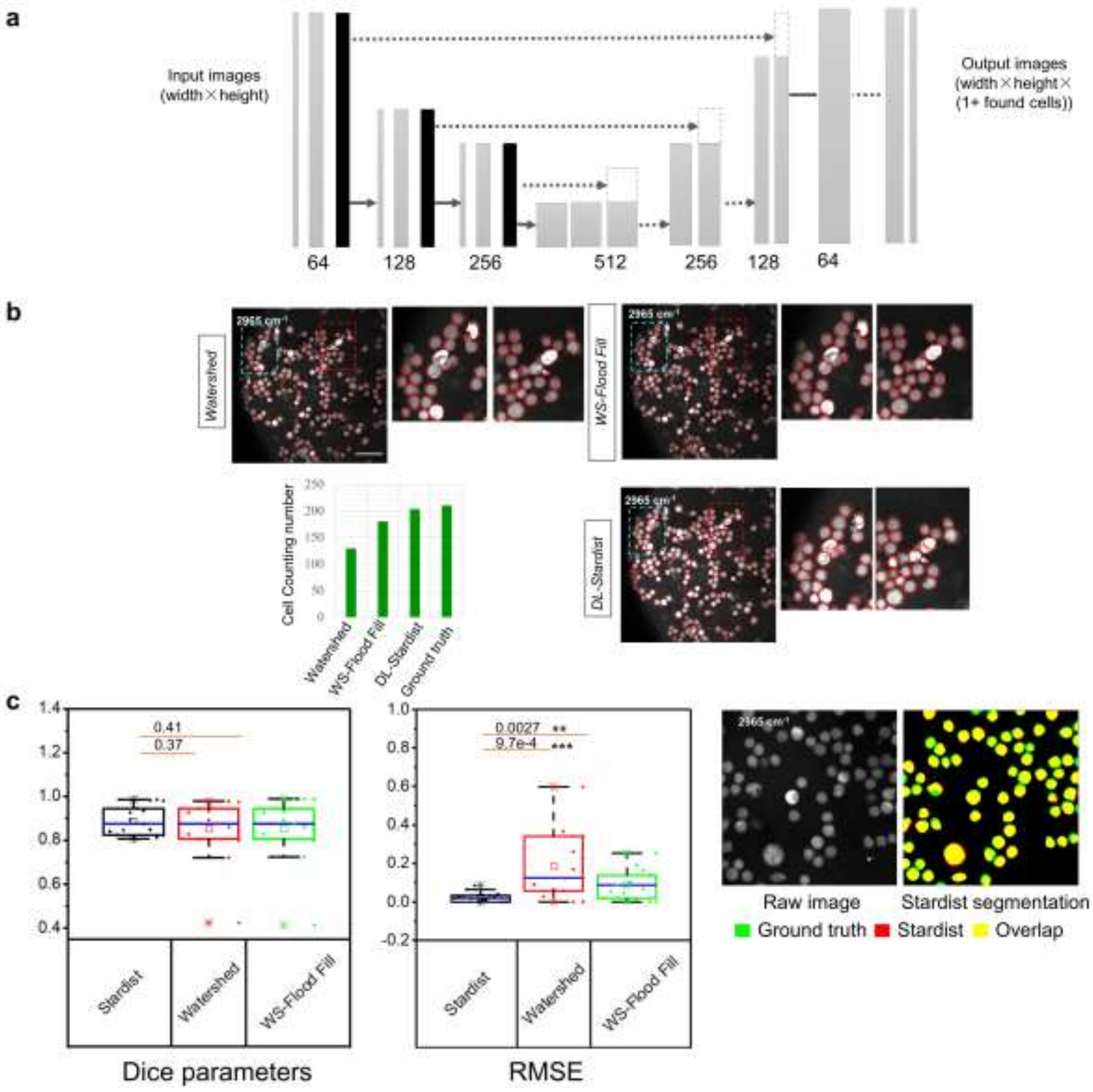

Fig. S1 | Performance of single cell segmentation by *stardist* model. a, Neural networks of *stardist* model. **b**, Typical segmentation results of watershed (WS) combing with fill flood method by using *imagej*, watershed by using standard *opencv* library, deep learning (DL) based *stardist* segmentation model. Cell count using watershed; WS-Flood Fill method; stardist; ground truth by manual visual judgment. Red line label the contours of cells, and two zoom-in regions. **c**, Dice parameter and relative root squared error (RRSE) between automated segmentation and ground truth (N=30), and cell segmentation overlay comparison between visual judgment and *stardist*. The box and whisker plots represent median values (center lines), mean values (horizontal bars), minimum and maximum (outliers), 25th to 75th percentiles (box edges) and 1.5x interquartile range (whiskers), with all points plotted. ***<0.0005, **<0.005, *<0.05, Scale bar: 20μm.

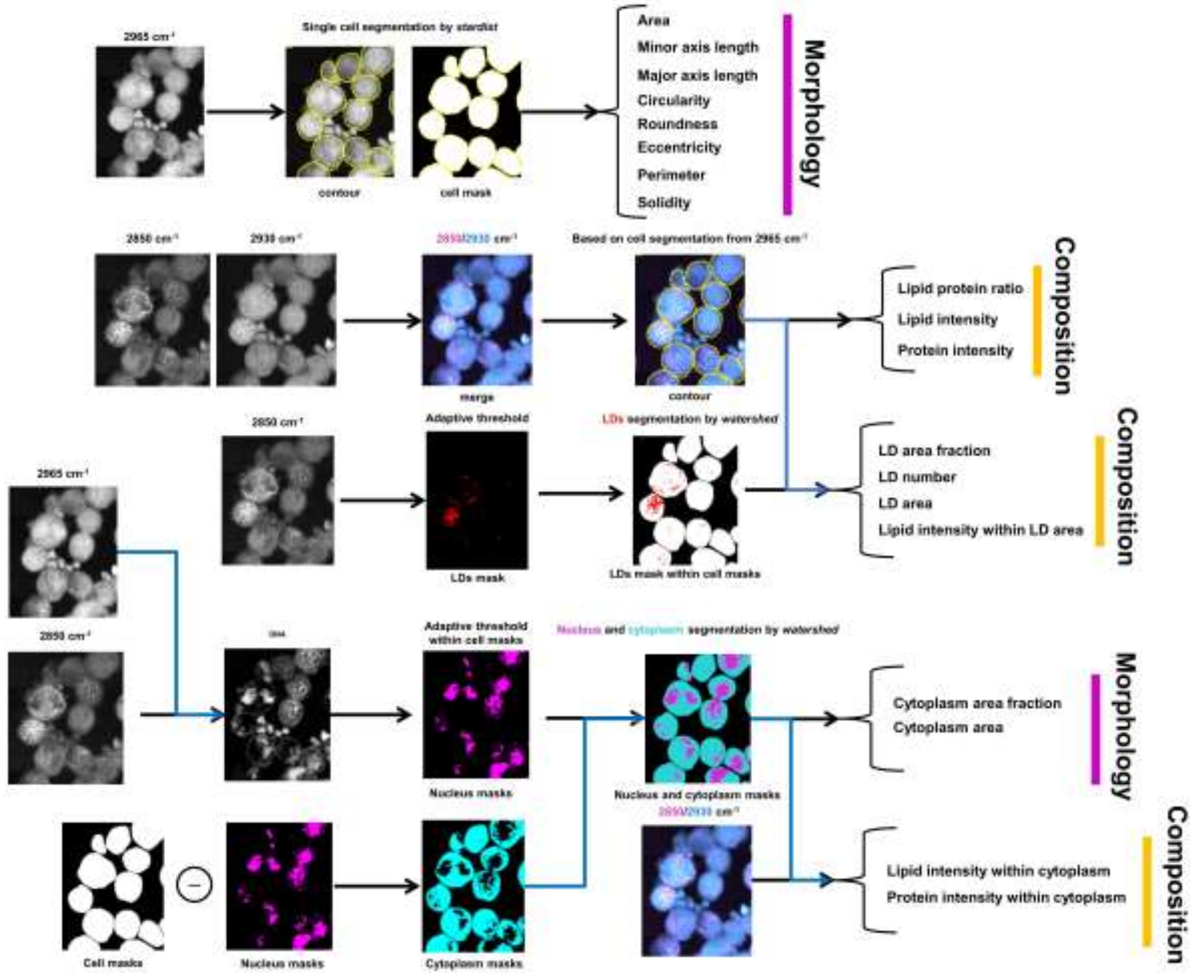

Fig. S2 | Workflow of feature extraction based on masks. Firstly, 8 morphology features extract from cell segmentation based on 2965cm^{-1} channel, and 3 composition features extract from 2850cm^{-1} and 2930cm^{-1} based on single cell segmentation mask. Finally, 6 composition features and 2 morphology features extracted from 2850cm^{-1} and 2930cm^{-1} based on single cell mask and LDs, nucleus and cytoplasm masks.

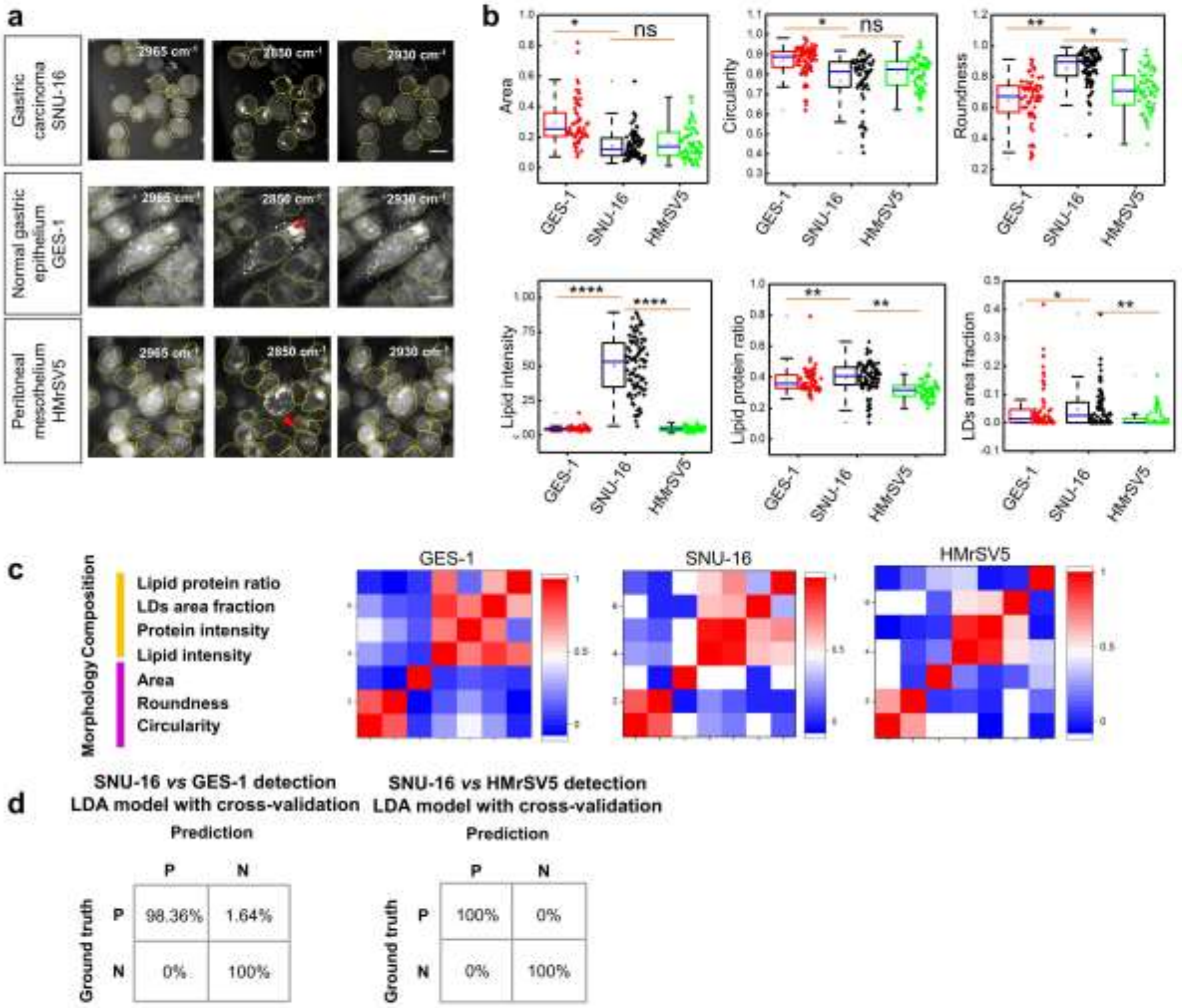

Fig. S3 | Results of SRMC of gastric cell lines with single cell segmentation and classification. a, Typical three-color SRS images of GES-1 (gastric epithelium cells), SNU-16 (differentiated carcinoma cells), and HMrSV5 (mesothelial cells). **b**, Three morphology features (area, roundness and circularity) and four composition features (lipid intensity, lipid protein ratio, LDs area fraction) comparisons of single cell between gastric cells. The box and whisker plots represent median values (center lines), mean values (horizontal bars), minimum and maximum (outliers), 25th to 75th percentiles (box edges) and 1.5x interquartile range (whiskers), with all points plotted. **c**, Correlation coefficient mapping about features with each other for gastric cells, **d**, confusion matrix of tumor cell detection by linear discriminate analysis (LDA) model. Scale bar: 20μm. ***<0.0005, **<0.005, *<0.05, ns: no significant difference.

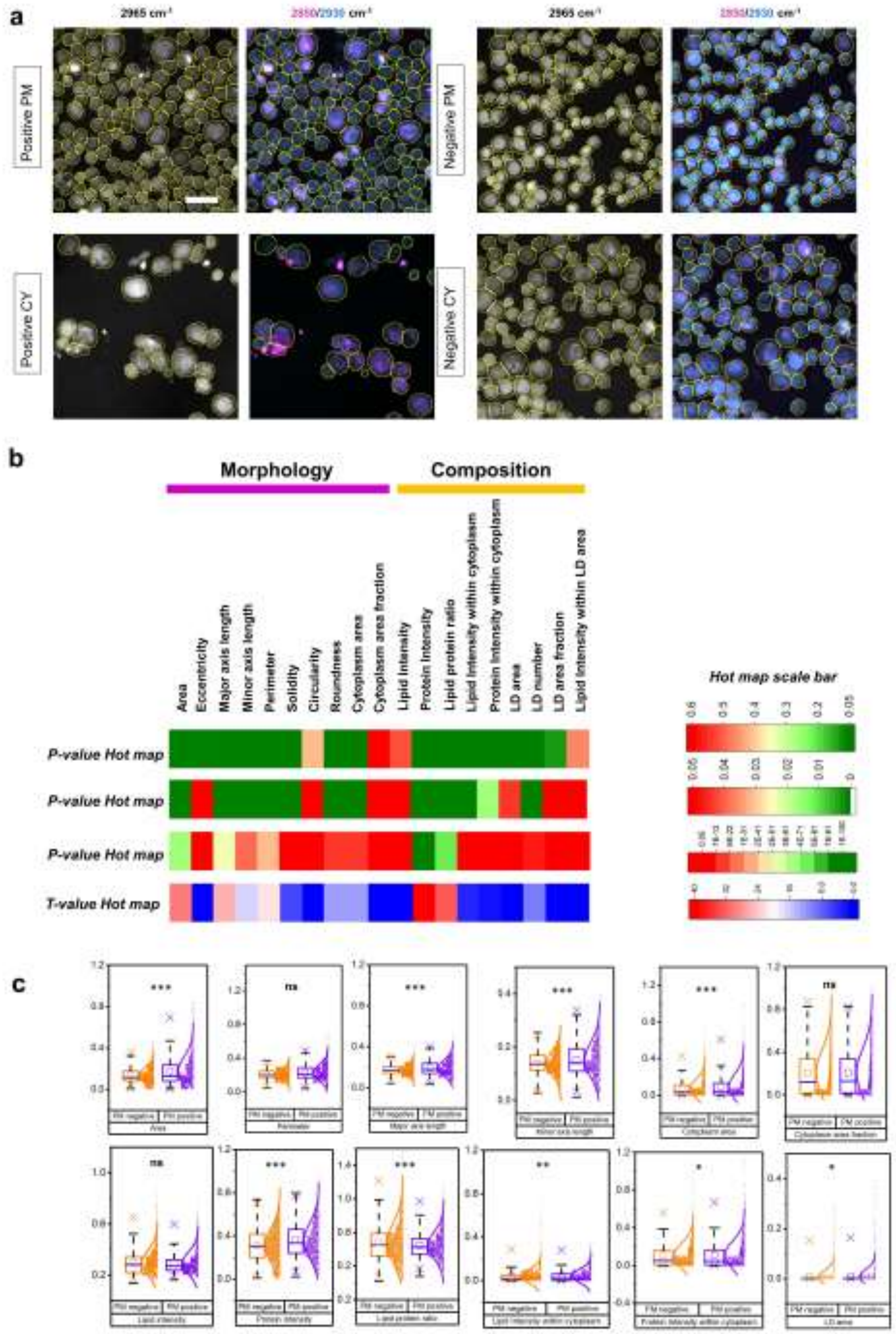

Fig. S4 | Representative SRS imaging of GC exfoliated cells and comparisons of 19 features between positive and negative PM. a, Typical images of PM positive and negative specimen. **b**, Hot map of p-values, and t-value by comparing PM positive and negative raw features with three different scale-bar, the features mainly have three types of morphology, composition, and lipid droplets (LD). **c**, feature comparisons between negative and positive PM. scale bar: 20μm. The box and whisker plots represent median values (center lines), mean values (horizontal bars), minimum and maximum (outliers), 25th to 75th percentiles (box edges) and 1.5x interquartile range (whiskers), with all points plotted. ***<0.0005, **<0.005, *<0.05, ns: no significant difference.

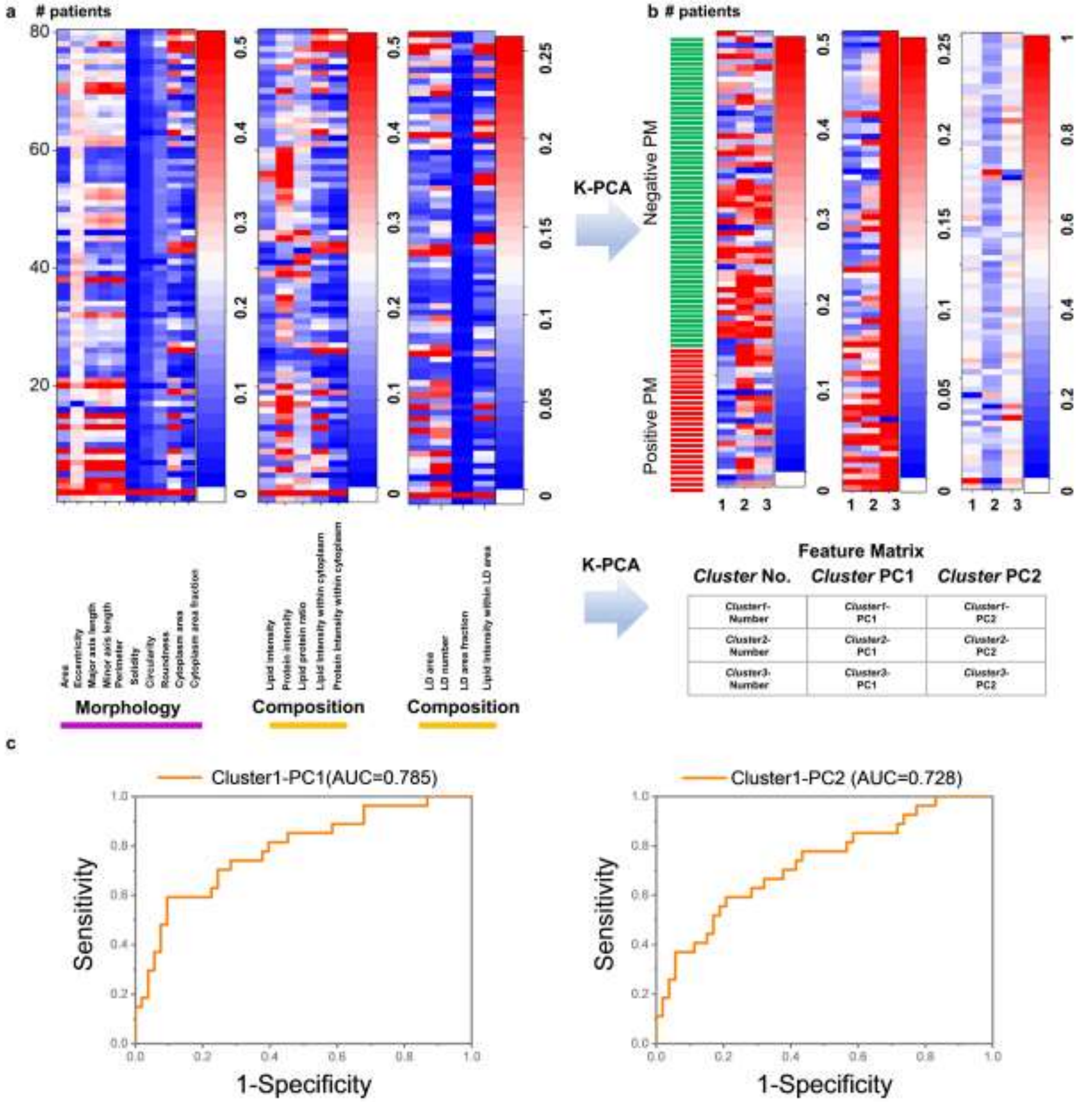

Fig. S5 | Hot map of raw feature and feature matrix transformed by K-PCA, and ROC analysis of feature matrix. a, 0-1 normalization of average values of raw features before K-PCA. **b**, 0-1 normalization of feature matrix after K-PCA based dimensional reduction. *Cluster1*-number and *Clutser1*-PC1 were obviously different between PM positive and negative. **c**, ROC curves of *Clutser1*-PC1 and *Clutser1*-PC2 for PM detection respectively.

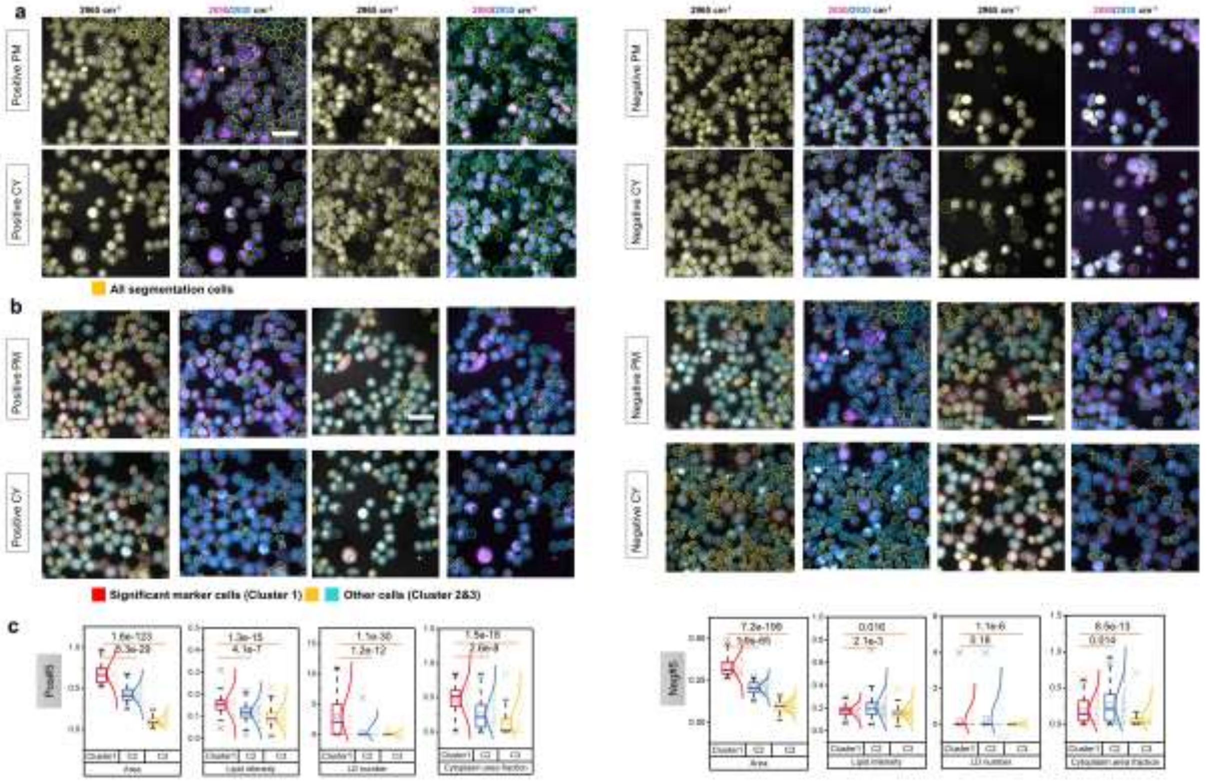

Fig. S6 | Representative images and quantitative analysis of exfoliated cell phenotyping. a, Typical cell segmentation results. Yellow labels the all contours. **b**, Typical cell phenotyping results by K-PCA. Red labels *Cluster 1*, cyan labels *Cluster 2* and yellow labels *Cluster 3*. **c**, Features (LD number, lipid intensity, cellular area and cytoplasm area fraction) quantification and comparisons among cell clusters in CY NEG/POS#5. The numbers with underlines denote *p* values. The box and whisker plots represent median values (center lines), mean values (horizontal bars), minimum and maximum (outliers), 25th to 75th percentiles (box edges) and 1.5x interquartile range (whiskers), with all points plotted.

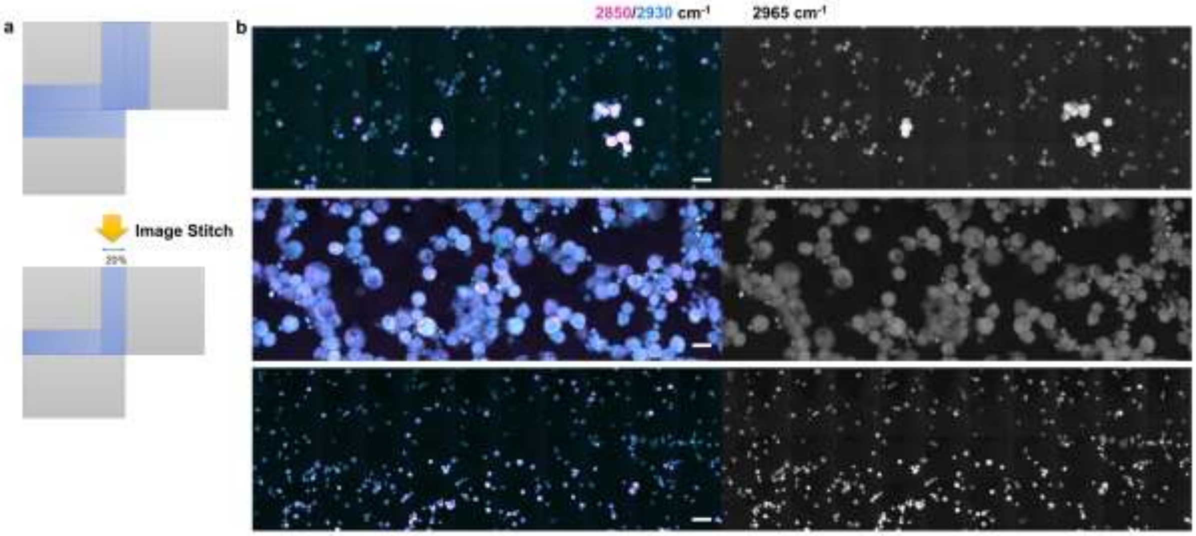

Fig. S7 | Image stitching of three-color SRS imaging of GC exfoliated cells. a, Schematic of image stitching, image tiles generated using the 20% overlap approach are stitched to an absolute Cartesian coordinate system. **b**, Typical three-color SRS imaging of GC exfoliated cells after image stitching. Upper shows typical cytology positive specimen with low percentage of tumor cells, and medium shows typical cytology specimen with high percentage of tumor cells, and lower shows typical cytology negative specimen without tumor cells. Scale bar: 20μm.

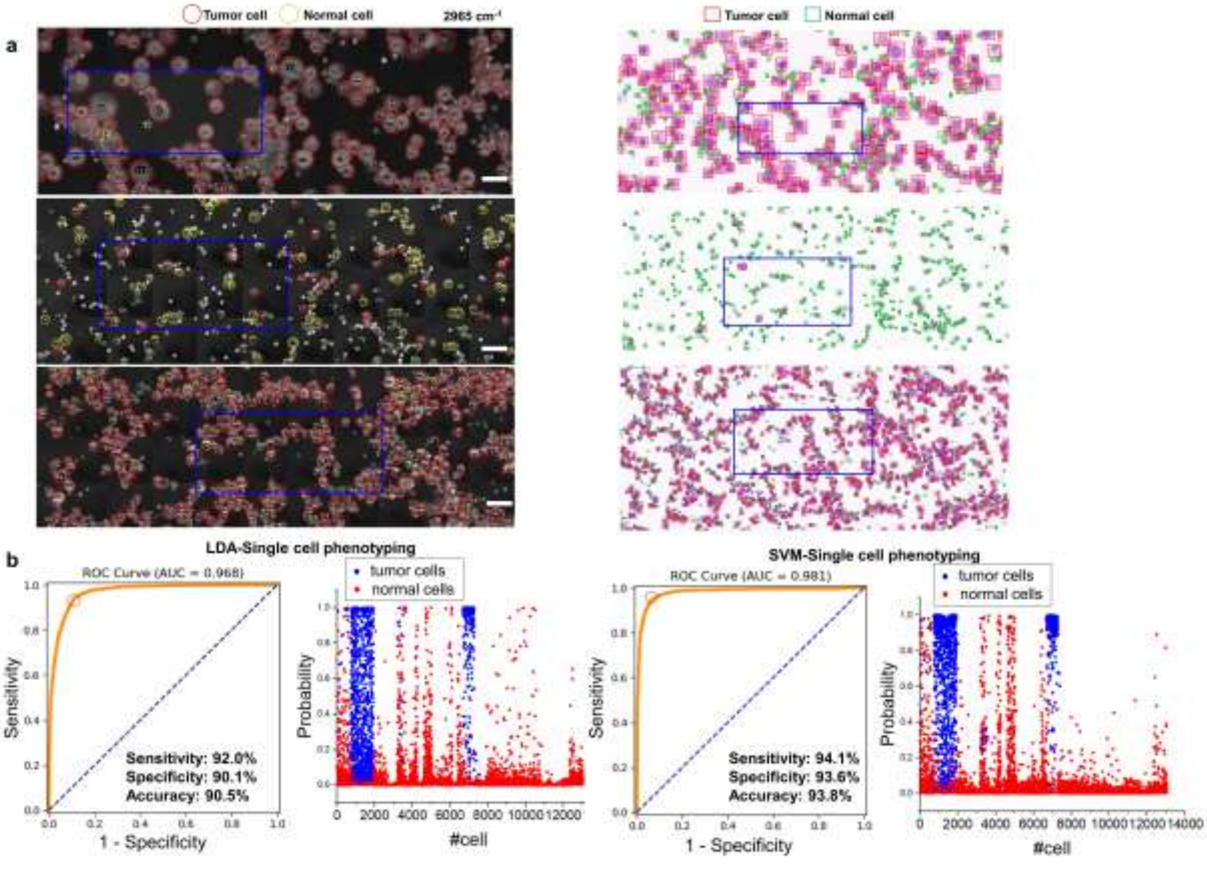

LDA model cross validation

Ground truth \ Prediction	T	N
T	1064	94
N	1146	10316

SVM model cross validation

Ground truth \ Prediction	T	N
T	1090	68
N	734	10728

Fig. S8 | In situ SRS&HE imaging of GC exfoliated cells. Results of single cell phenotyping by machine learning (ML) classifiers (SVM and LDA). **a**, Typical H&E&SRS images of GC cells with cell phenotyping results. We collected imaging data to create the training and test dataset include GC exfoliated cells (1158 normal cells; 11462 tumor cells). **b**, ROC curve (left), the AUCs, sensitivity, specificity and accuracy and the positive probability of cell phenotyping (right), using ML algorithms. **c**, Confusion matric of single cell phenotyping using ML algorithms.

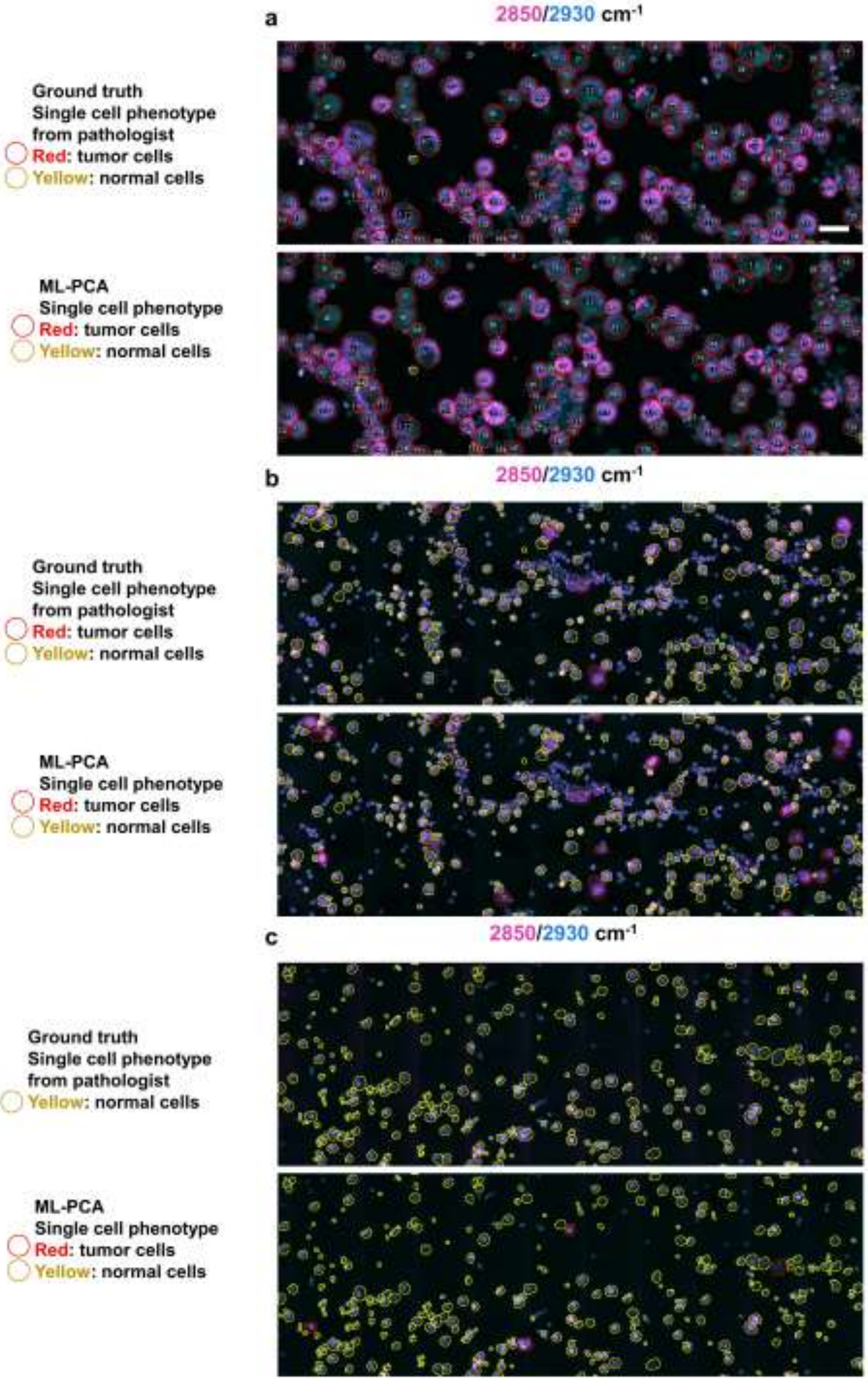

Fig. S9 | Typical tumor cell and normal cell detection by ML-PCA. Comparisons of ML-PCA results and H&E results for three types of specimens. SRS&HE in situ images were labeled by two senior pathologists. **a**, Typical PM positive specimen with high percentage of tumor cells. **b**, Typical PM positive specimen with low percentage of tumor cells. **c**, Typical PM negative specimen without tumor cells.

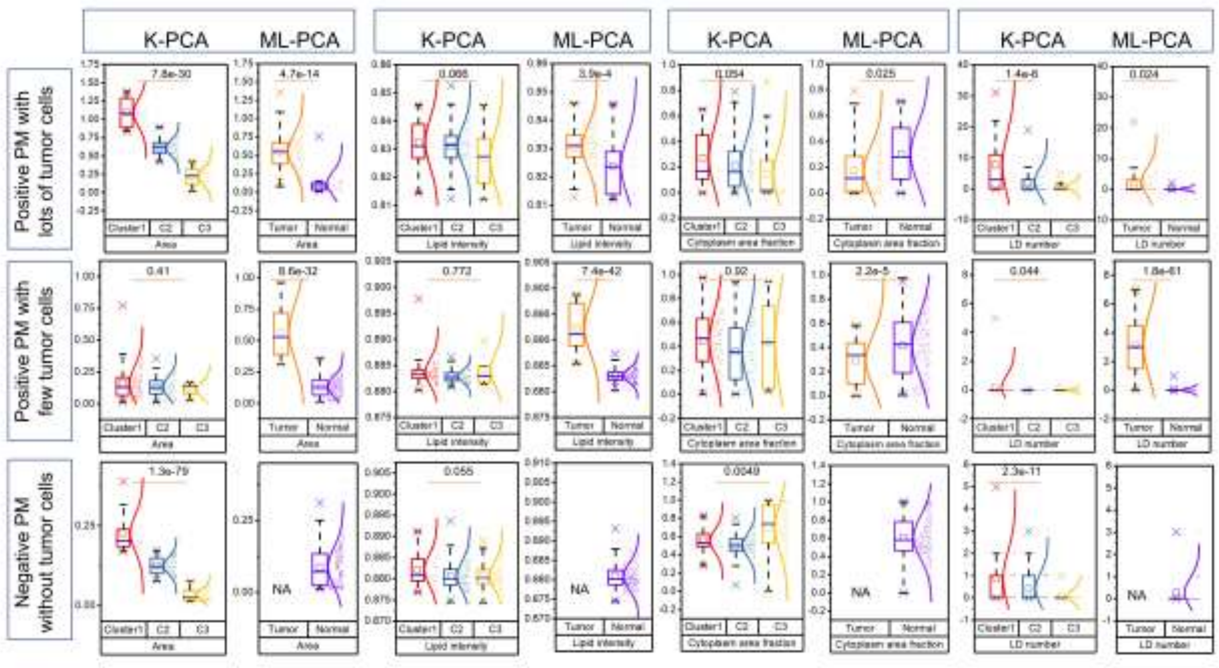

Fig. S10 | Results of feature profiles by K-PCA and ML-PCA. We compared the features including area, lipid intensity, LD number, cytoplasm area fraction of three typical specimen with p-value. PM positive specimen with high percentage of tumor cells. Typical PM positive specimen with low percentage of tumor cells. Typical PM negative specimen without tumor cells. The box and whisker plots represent median values (center lines), mean values (horizontal bars), minimum and maximum (outliers), 25th to 75th percentiles (box edges) and 1.5x interquartile range (whiskers), with all points plotted.

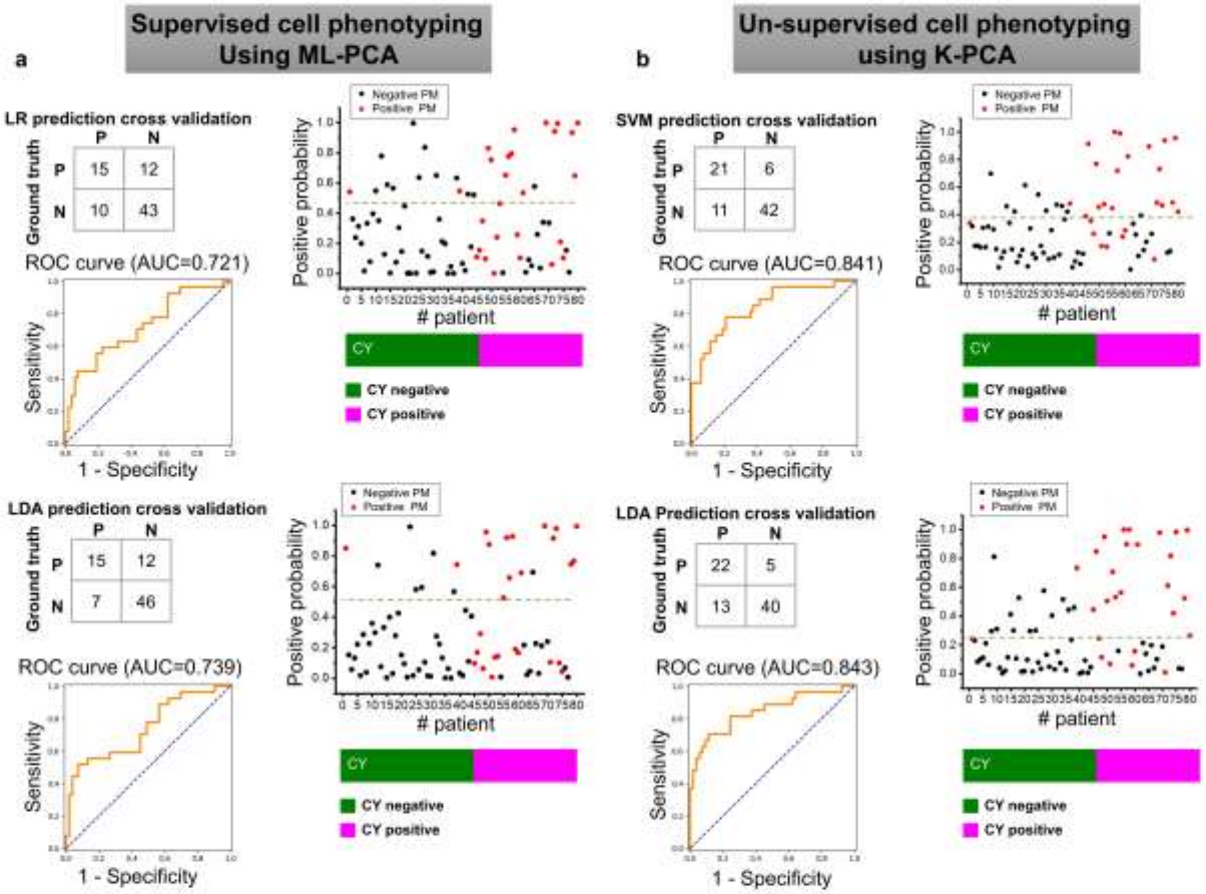

Fig. S11 | Results of PM detection by classifiers (SVM, LDA, LR etc.). The input feature matrix respectively comes from supervised ML-PCA and unsupervised K-PCA cell phenotyping methods. PM positive probability with cross-validation, confusion matrix and ROC curves of 80 patients (27 positive PM; 53 negative PM, 35 positive cytology; 45 negative cytology). **a**, Using ML-PCA algorithm, the AUCs were respectively 0.797, 0.739 and 0.721 by SVM, LDA, and LR. The best performance of ML-PCA and SVM model was 0.797 described in **Fig. 4**, ML-PCA and LR/LDA was here. **b**, Using K-PCA algorithm, the AUCs were respectively 0.841, 0.85 and 0.843 by SVM, LDA, and LR. The best performance of K-PCA and LR was 0.85 described in **Fig. 4**, K-PCA and SVM/LDA was here.

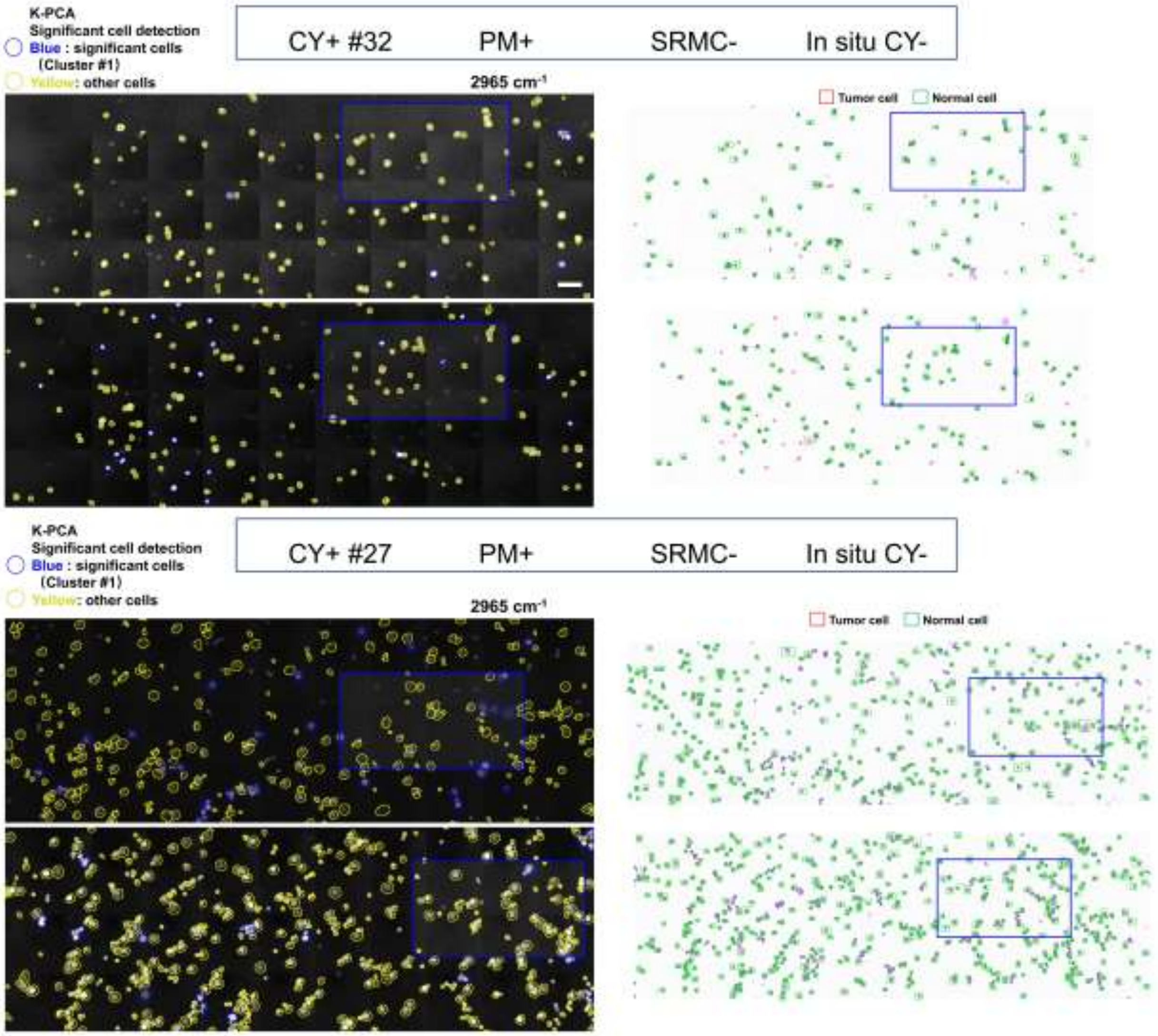

Fig. S12 | Typical SRS&HE in-situ images for SRMC negative detection. The mismatch between CY+, PM+ and our SRMC- method.

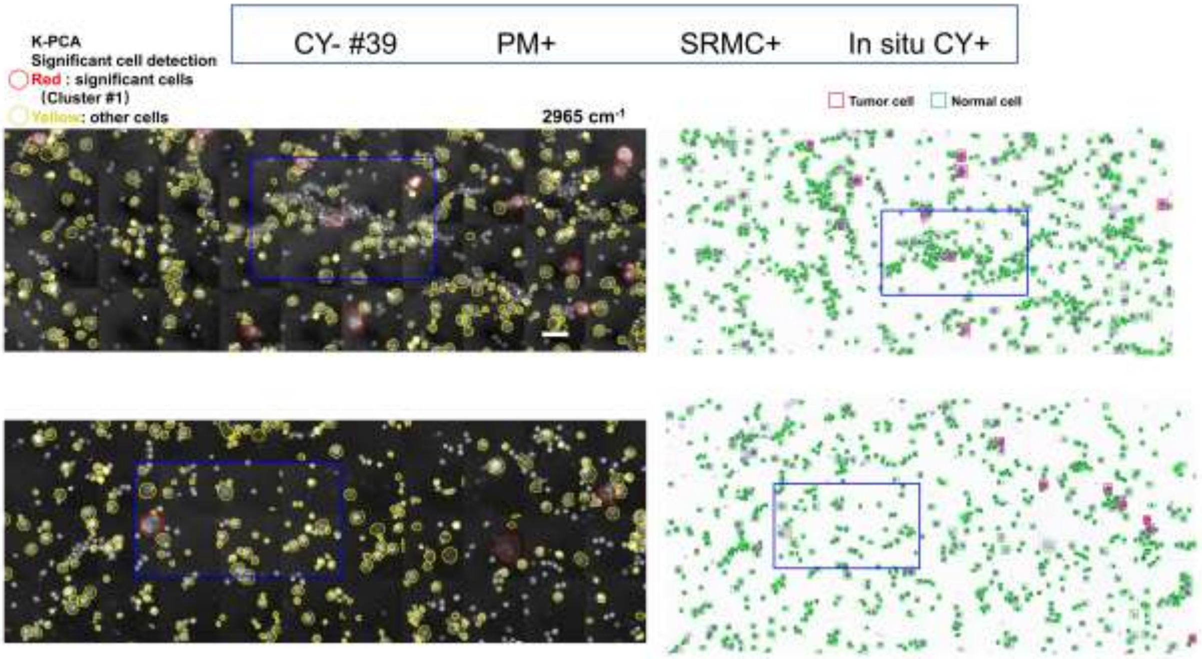

Fig. S13 | Typical SRS&HE in-situ images for SRMC positive detection. The mismatch between CY-, PM+ and our SRMC+ method.

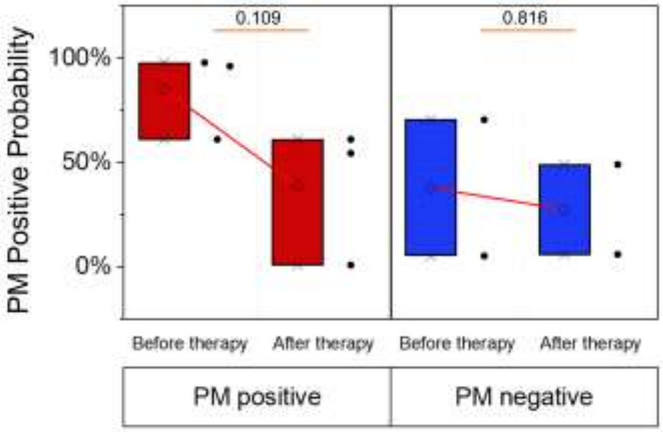

Fig. S14 | PM positive probability before and after chemotherapy for patients diagnosed with PM positive (n = 3) and PM negative (n=2). The numbers with underlines denote *p* values. The box and whisker plots represent median values (center lines), mean values (horizontal bars), minimum and maximum (outliers), 25th to 75th percentiles (box edges) and 1.5x interquartile range (whiskers), with all points plotted.

Table S1 | Typical raw features with 0-1 normalization from 80 patients.

Patient ID	Conventional Cytology results	Histopathology results	Area	Lipid intensity	Protein intensity	Cytoplasm area fraction	LD number
NEG#1*	negative	positive	0.192	0.114	0.285	0.027	0.073
NEG#2	negative	negative	0.488	1	0.947	1	1
NEG#3	negative	negative	0.385	0.165	0.387	0.066	0.206
NEG#4	negative	negative	0.460	0.171	0.251	0.047	0.306
NEG#5	negative	negative	0.049	0.072	0.177	0.004	0.014
NEG#6	negative	negative	0.619	0.316	0.694	0.084	0.263
NEG#7	negative	negative	0.774	0.083	0.095	0.010	0.410
NEG#8	negative	negative	0.158	0.1116	0.357	0.181	0.086
NEG#9	negative	negative	0.694	0.326	0.747	0.125	0.441
NEG#10	negative	negative	0.056	0.093	0.212	0.008	0.042
NEG#11	negative	negative	0.155	0.152	0.364	0.028	0.109
NEG#12	negative	negative	0.164	0.167	0.469	0.048	0.194
NEG#13	negative	negative	1	0.045	0.054	0.064	0.192
NEG#14	negative	negative	0.151	0.158	0.456	0.018	0.143
NEG#15	negative	negative	0.877	0.160	0.395	0.157	0.089
NEG#16	negative	negative	0.025	0.251	0.706	0.030	0.044
NEG#17	negative	negative	0.157	0.392	0.664	0.170	0.178
NEG#18	negative	negative	0.192	0.242	0.588	0.058	0.073
NEG#19	negative	negative	0.175	0.098	0.304	0.376	0.221
NEG#20	negative	negative	0.499	0.313	0.700	0.002	0.206
NEG#21	negative	negative	0.375	0.076	0.196	0.084	0.218
NEG#22	negative	negative	0.039	0.127	0.320	0.021	0.047
NEG#23	negative	negative	0.069	0.067	0.213	0.007	0.040
NEG#24	negative	negative	0.118	0.054	0.141	0.025	0.030
NEG#25	negative	negative	0.109	0.021	0.060	0.039	0.124
NEG#26	negative	negative	0.114	0.015	0.098	0.531	0.350
NEG#27	negative	negative	0.151	0.096	0.146	0.238	0.222
NEG#28	negative	negative	0.169	0.116	0.363	0.044	0.051
NEG#29	negative	negative	0.125	0.083	0.269	0.041	0.060
NEG#30	negative	negative	0.152	0.119	0.349	0.174	0.111
NEG#31	negative	negative	0.148	0.093	0.325	0.150	0.152
NEG#32	negative	negative	0.283	0.049	0.076	0.037	0.202
NEG#33	negative	negative	0.093	0.107	0.381	0	0.026
NEG#34	negative	negative	0.181	0.126	0.415	0.067	0.142
NEG#35	negative	negative	0.048	0.164	0.500	0.008	0.041
NEG#36	negative	negative	0.037	0.096	0.264	0.033	0.025
NEG#37	negative	negative	0.038	0.099	0.215	0.023	0.030
NEG#38	negative	negative	0.430	0.104	0.142	0.033	0.037
NEG#39*	negative	positive	0.073	0.328	0.495	0.080	0.067
NEG#40	negative	negative	0.133	0.140	0.417	0.028	0.060
NEG#41	negative	negative	0.066	0	0	0.132	0.130
NEG#42	negative	negative	0.102	0.071	0.146	0.040	0.098
NEG#43	negative	negative	0.066	0.021	0.156	0.455	0.296
NEG#44	negative	negative	0.04	0.077	0.161	0.484	0.402
NEG#45	negative	negative	0.215	0.283	0.507	0.103	0.046
POS#1	positive	positive	0	0.284	0.337	0.250	0.105
POS#2	positive	positive	0.222	0.098	0.282	0.013	0.129
POS#3	positive	positive	0.260	0.109	0.218	0.059	0.139
POS#4	positive	positive	0.227	0.136	0.274	0.018	0.02

POS#5	positive	positive	0.091	0.107	0.315	0.008	0.040
POS#6	positive	positive	0.190	0.142	0.378	0.025	0.091
POS#7	positive	positive	0.256	0.196	0.436	0.022	0.077
POS#8	positive	positive	0.231	0.170	0.379	0.033	0.120
POS#9	positive	positive	0.173	0.180	0.508	0.141	0.186
POS#10*	positive	negative	0.045	0.384	1	0.074	0
POS#11	positive	positive	0.063	0.465	0.975	0.133	0.029
POS#12	positive	positive	0.067	0.171	0.511	0.064	0.057
POS#13	positive	positive	0.048	0.202	0.550	0.168	0.044
POS#14	positive	positive	0.041	0.273	0.795	0.035	0.061
POS#15	positive	positive	0.036	0.214	0.470	0.028	0
POS#16	positive	positive	0.289	0.115	0.161	0.168	0.157
POS#17	positive	positive	0.218	0.115	0.269	0.011	0.128
POS#18*	positive	negative	0.175	0.109	0.184	0.282	0.248
POS#19*	positive	negative	0.143	0.165	0.313	0.178	0.085
POS#20*	positive	negative	0.185	0.174	0.256	0.086	0.099
POS#21*	positive	negative	0.133	0.128	0.270	0.218	0.177
POS#22*	positive	negative	0.144	0.087	0.154	0.053	0.034
POS#23*	positive	negative	0.175	0.126	0.348	0.056	0.068
POS#24*	positive	negative	0.135	0.052	0.155	0.014	0.029
POS#25	positive	positive	0.394	0.182	0.528	0.142	0.566
POS#26*	positive	negative	0.367	0.076	0.190	0.106	0.189
POS#27	positive	positive	0.191	0.084	0.200	0.024	0.105
POS#28	positive	positive	0.190	0.079	0.104	0.294	0.122
POS#29	positive	positive	0.088	0.129	0.395	0.021	0.031
POS#30	positive	positive	0.217	0.187	0.249	0.234	0.162
POS#31	positive	positive	0.180	0.091	0.166	0.085	0.102
POS#32*	positive	negative	0.076	0.188	0.222	0.483	0.135
POS#33	positive	positive	0.290	0.081	0.191	0.478	0.665
POS#34	positive	positive	0.141	0.101	0.279	0.265	0.087
POS#35	positive	positive	0.157	0.076	0.125	0.405	0.437

* represents the mismatch between cytological and histopathological results.

Table S2 | Values of *Cluster1*-PC1, *Cluster1*-PC2 and *Cluster2*-PC1, *Cluster2*-PC2 of 80 patients.

Patient ID	Cytological results	Laparoscopic results	[a]C1	[b]C2	[c]C3	Cluster1-PC1	Cluster2-PC1	Cluster1-PC2	Cluster2-PC2
NEG#1*	negative	positive	29	180	306	1737.094	408.314	173.373	-76.369
NEG#2	negative	negative	51	296	245	844.614	89.835	-7.123	-26.401
NEG#3	negative	negative	18	34	6	445.168	-117.110	-24.275	19.424
NEG#4	negative	negative	43	86	95	938.398	150.506	-10.108	-4.268
NEG#5	negative	negative	87	198	408	708.001	227.756	-59.817	60.633
NEG#6	negative	negative	35	181	194	1158.821	180.735	-15.841	-20.370
NEG#7	negative	negative	66	115	192	1027.32	215.410	27.431	-43.363
NEG#8	negative	negative	87	159	207	957.964	240.060	-117.903	70.044
NEG#9	negative	negative	37	468	227	902.757	84.886	102.554	-16.743
NEG#10	negative	negative	50	82	184	1225.35	424.159	-103.639	80.883
NEG#11	negative	negative	81	219	331	757.296	101.472	56.199	-46.566
NEG#12	negative	negative	208	321	298	457.147	27.520	25.156	-24.349
NEG#13	negative	negative	32	93	194	797.009	143.337	-1.691	-22.385
NEG#14	negative	negative	100	205	517	666.129	180.469	-76.770	51.629
NEG#15	negative	negative	41	8	32	260.560	-80.291	-24.717	670.329
NEG#16	negative	negative	16	109	178	1284.856	-46.309	-37.757	381.138
NEG#17	negative	negative	98	285	367	715.981	237.612	94.674	-30.277
NEG#18	negative	negative	113	388	349	729.73	227.736	7.153	1.716
NEG#19	negative	negative	117	154	479	821.525	252.443	100.075	-79.735
NEG#20	negative	negative	152	229	312	478.623	111.957	-75.776	50.604
NEG#21	negative	negative	75	188	421	974.253	353.495	104.500	-40.222
NEG#22	negative	negative	21	170	437	1520.546	561.952	208.092	-98.595
NEG#23	negative	negative	132	155	369	816.703	213.091	54.045	-58.634
NEG#24	negative	negative	3	16	126	1777.592	720.063	-280.841	89.608
NEG#25	negative	negative	154	267	210	481.863	96.590	-152.683	133.818
NEG#26	negative	negative	94	178	552	739.063	330.870	110.313	-107.104
NEG#27	negative	negative	7	89	158	2378.29	435.110	-97.483	1.340
NEG#28	negative	negative	135	218	420	823.981	231.311	-84.849	84.967
NEG#29	negative	negative	112	336	283	664.221	9.947	54.300	-50.059
NEG#30	negative	negative	28	137	421	1320.432	488.849	-61.390	-6.251
NEG#31	negative	negative	119	212	297	732.102	-20.216	-49.950	84.793
NEG#32	negative	negative	59	255	140	920.558	61.997	113.268	-54.742
NEG#33	negative	negative	127	198	514	763.668	268.196	-26.423	32.675
NEG#34	negative	negative	53	253	359	1126.836	361.082	26.231	-9.368
NEG#35	negative	negative	122	249	285	533.805	113.539	6.119	0.049
NEG#36	negative	negative	66	315	475	870.815	91.880	-66.679	42.088
NEG#37	negative	negative	77	271	555	904.256	144.142	36.072	-24.010
NEG#38	negative	negative	78	198	177	1301.845	173.017	46.695	-57.655
NEG#39*	negative	positive	116	325	601	1509.39	308.400	-35.687	28.320
NEG#40	negative	negative	179	101	132	476.672	-60.531	2.140	-28.408
NEG#41	negative	negative	184	151	313	685.154	23.928	-69.651	161.737
NEG#42	negative	negative	152	378	397	692.580	131.260	72.172	-59.008
NEG#43	negative	negative	90	100	105	518.024	-64.426	-6.299	11.182
NEG#44	negative	negative	27	123	195	870.637	128.515	-21.545	16.332
NEG#45	negative	negative	131	222	307	732.973	211.145	48.172	-35.386
POS#1	positive	positive	15	277	304	1351.002	217.660	45.233	-12.268
POS#2	positive	positive	14	377	329	2236.688	158.635	75.074	-51.025
POS#3	positive	positive	46	338	558	965.951	230.955	203.012	-68.181
POS#4	positive	positive	26	264	349	893.065	174.681	123.538	-30.362

POS#5	positive	positive	46	108	201	1950.394	580.662	-257.156	195.820
POS#6	positive	positive	44	139	153	1514.125	343.351	24.778	-18.591
POS#7	positive	positive	46	214	96	808.706	15.660	5.539	-6.503
POS#8	positive	positive	53	380	232	1129.354	114.743	-92.609	22.080
POS#9	positive	positive	1	4	34	1631.954	781.733	-833.677	312.455
POS#10*	positive	negative	46	243	125	733.400	70.399	0.751	-17.881
POS#11	positive	positive	37	242	387	1211.185	248.607	-116.690	54.805
POS#12	positive	positive	7	154	354	6433.348	1047.681	50.451	-315.115
POS#13	positive	positive	14	173	393	1942.708	557.735	-253.926	62.929
POS#14	positive	positive	34	137	78	3089.619	46.630	58.197	-144.437
POS#15	positive	positive	11	15	80	905.032	644.500	-322.444	232.145
POS#16	positive	positive	63	216	280	1079.778	196.359	23.834	-25.271
POS#17	positive	positive	19	142	831	2114.345	685.251	-71.387	59.997
POS#18*	positive	negative	251	219	261	531.640	-83.896	29.986	-74.706
POS#19*	positive	negative	38	104	72	953.422	114.940	-6.245	-6.744
POS#20*	positive	negative	24	134	92	929.428	125.100	-46.843	8.561
POS#21*	positive	negative	38	78	55	827.804	-67.098	99.496	-139.662
POS#22*	positive	negative	5	35	121	1980.221	594.470	-99.397	7.341
POS#23*	positive	negative	27	143	100	894.292	106.843	153.310	-71.751
POS#24*	positive	negative	92	227	296	948.777	57.226	-26.856	39.538
POS#25	positive	positive	41	92	123	2243.022	504.56	-104.258	84.040
POS#26*	positive	negative	90	85	160	929.046	238.233	-95.467	138.634
POS#27	positive	positive	112	145	303	754.143	113.493	-12.433	26.555
POS#28	positive	positive	52	68	71	1590.997	62.701	65.302	-95.256
POS#29	positive	positive	34	106	124	2152.295	513.800	123.822	-69.771
POS#30	positive	positive	19	437	267	1038.345	81.668	100.223	-18.534
POS#31	positive	positive	22	304	247	2356.749	326.469	-145.170	4.644
POS#32*	positive	negative	75	186	152	662.761	41.833	8.090	-3.524
POS#33	positive	positive	61	184	183	1409.766	262.881	8.388	-1.547
POS#34	positive	positive	3	35	104	8111.398	2180.946	878.87	-282.361
POS#35	positive	positive	13	44	161	2665.016	587.554	23.094	13.085

* represents the mismatch between the cytological and histopathological results.

[a]C1: Cluster1 cell number, [b]C2: Cluster2 cell number, [c]C3: Cluster3 cell number.

Table S3 | Diagnostic results for each patient, including conventional cytology, histopathology, and SRMC (based on SVM, LDA, or LR models) predicted probability of PM.

Patient ID	Cytological results	Laparoscopic results	age	sex	SRMC (SVM) positive probability (%)	SRMC (LDA) positive probability (%)	SRMC (LR) positive probability (%)
NEG#1*	negative	positive	60	male	33.77	28.54	43.96
NEG#2	negative	negative	57	male	34.94	39.68	29.29
NEG#3	negative	negative	42	male	15.34	0.77	10.61
NEG#4	negative	negative	62	male	20.97	0.90	12.62
NEG#5	negative	negative	70	female	20.23	7.49	8.21
NEG#6	negative	negative	72	female	30.91	27.28	32.13
NEG#7	negative	negative	52	male	13.01	2.96	6.26
NEG#8	negative	negative	44	female	31.66	39.19	22.13
NEG#9	negative	negative	60	male	66.44	90.85	90.86
NEG#10	negative	negative	59	male	31.85	10.73	33.42
NEG#11	negative	negative	63	male	10.98	3.90	3.50
NEG#12	negative	negative	74	male	4.27	0.89	0.05
NEG#13	negative	negative	64	male	11.35	1.84	3.66
NEG#14	negative	negative	52	male	15.01	14.42	9.61
NEG#15	negative	negative	63	male	44.87	98.41	99.62
NEG#16	negative	negative	48	male	35.46	99.95	16.44
NEG#17	negative	negative	59	male	17.93	5.46	5.37
NEG#18	negative	negative	47	male	48.60	56.37	25.17
NEG#19	negative	negative	68	male	3.28	0.80	0.76
NEG#20	negative	negative	57	male	6.12	0.10	0.46
NEG#21	negative	negative	51	female	17.07	12.40	8.54
NEG#22	negative	negative	45	female	57.86	37.22	64.01
NEG#23	negative	negative	58	male	6.04	0.73	0.53
NEG#24	negative	negative	27	male	30.77	16.88	35.36
NEG#25	negative	negative	54	male	9.62	2.72	0.90
NEG#26	negative	negative	58	male	8.16	2.75	2.59
NEG#27	negative	negative	36	female	60.93	70.62	90.54
NEG#28	negative	negative	50	male	18.53	9.34	5.66
NEG#29	negative	negative	60	male	12.47	4.44	2.88
NEG#30	negative	negative	67	male	42.86	67.78	59.50
NEG#31	negative	negative	54	male	10.23	1.01	1.31
NEG#32	negative	negative	65	male	24.62	29.67	23.18
NEG#33	negative	negative	50	male	14.70	7.76	3.83
NEG#34	negative	negative	36	female	47.34	43.99	55.12
NEG#35	negative	negative	77	male	7.39	2.71	0.86
NEG#36	negative	negative	28	female	49.03	34.90	50.26
NEG#37	negative	negative	71	female	35.30	20.02	31.29
NEG#38	negative	negative	39	female	39.79	55.14	58.86
NEG#39*	negative	positive	66	female	47.13	77.83	64.23
NEG#40	negative	negative	67	male	0.65	2.93	0.04
NEG#41	negative	negative	63	male	14.18	0.37	0.29
NEG#42	negative	negative	39	female	14.25	4.38	2.72
NEG#43	negative	negative	48	male	7.09	0.35	0.42
NEG#44	negative	negative	61	male	13.39	2.87	6.93
NEG#45	negative	negative	69	male	7.74	2.69	1.19
POS#1	positive	positive	34	female	44.56	46.99	67.27

POS#2	positive	positive	61	female	79.69	59.56	97.61
POS#3	positive	positive	56	female	34.39	17.01	44.99
POS#4	positive	positive	57	female	24.04	10.30	23.14
POS#5	positive	positive	34	female	88.49	96.69	95.84
POS#6	positive	positive	55	male	39.32	49.76	57.65
POS#7	positive	positive	68	male	17.55	22.39	7.55
POS#8	positive	positive	55	male	53.37	84.53	63.11
POS#9	positive	positive	61	male	18.66	24.20	72.38
POS#10*	positive	negative	54	male	24.10	1.74	20.23
POS#11	positive	positive	61	male	48.78	72.27	61.81
POS#12	positive	positive	61	female	98.98	99.99	99.98
POS#13	positive	positive	56	male	65.83	88.58	94.11
POS#14	positive	positive	60	male	97.00	99.99	99.99
POS#15	positive	positive	57	male	22.42	4.69	6.76
POS#16	positive	positive	61	male	28.02	7.09	17.17
POS#17	positive	positive	60	male	76.48	91.58	98.88
POS#18*	positive	negative	67	male	0.17	0	0
POS#19*	positive	negative	68	male	29.32	2.15	29.92
POS#20*	positive	negative	64	male	23.80	2.77	18.03
POS#21*	positive	negative	40	male	10.86	5.32	15.24
POS#22*	positive	negative	67	male	36.89	87.28	31.25
POS#23*	positive	negative	56	male	12.58	5.13	11.20
POS#24*	positive	negative	69	male	16.28	3.54	6.86
POS#25	positive	positive	58	male	93.19	99.49	98.44
POS#26*	positive	negative	61	male	24.67	40.20	14.52
POS#27	positive	positive	57	male	3.41	0.65	0.14
POS#28	positive	positive	63	male	47.99	68.72	84.00
POS#29	positive	positive	74	male	71.98	87.67	91.69
POS#30	positive	positive	51	female	47.74	35.69	66.47
POS#31	positive	positive	38	female	94.92	98.31	99.26
POS#32*	positive	negative	38	female	8.13	2.83	2.37
POS#33	positive	positive	75	female	43.54	52.07	51.96
POS#34	positive	positive	55	male	94.9	99.02	99.86
POS#35	positive	positive	35	female	39.19	8.41	38.33

* represents the mismatch between cytological and histopathological results.

Table S4 | Performance comparisons between K-PCA and ML-PCA methods.

Phenotyping methods	Diagnostic models	Sensitivity	Specificity	Accuracy	NPV[a]	PPV[b]
K-PCA	LR	81.4%	85.0%	83.75%	90%	73.3%
	SVM	77.1%	80.4%	78.75%	87.5%	65.6%
	LDA	81.4%	75.5%	77.5%	88.8%	62.8%
ML-PCA	LR	62.9%	88.7%	80%	82.5%	73.9%
	SVM	55.5%	81.14%	72.5%	78.2%	60%
	LDA	55.5%	86.8%	76.25%	79.3%	68.2%

[a]NPV: negative predictive value, [b]PPV: positive predictive value.

Table S5 | Comparisons between our SRMC results and conventional cytology, histopathology, respectively.

**Conventional Cytology**[a, b]	**Histopathology**[a, b]	**SRMC**[a, b]	**Number of patients**
CY+	PM+	SRMC+	20
CY+	PM+	SRMC-	5 (false negative) (see Fig. S12)
CY+	PM-	SRMC+	0
CY+	PM-	SRMC-	10
CY-	PM+	SRMC+	2 (see Fig. S13)
CY-	PM+	SRMC-	0
CY-	PM-	SRMC+	8 (false positive)
CY-	PM-	SRMC-	35

[a]+: positive, [b]-: negative.

Table S6 | Image acquisition and processing workflow by task, software, and description.

	Task	Commercial (software) or custom (Github code name)	Description	Contributions / improvements
Imaging acquisition and stitching	Image acquisition with XY mapping	Commercial *Sciscan* 1.0 by LabVIEW	Public source code for large-area scanning imaging	Implement XY mapping with customized stage
	Image stiching with mapping to absolute coordinate system	Customized program by *MATLAB*	Seamless stitching of image tiles into a large FOV	Import variable overlay height and width pixel numbers when stitching large FOV
Single cell analysis	Cell segmentation	Commercial *Stardist* model facilitated by python TensorFlow library	Public source code for H&E or dye-staining cell segmentation	Pre-processing and post processing optimization for multicolor SRS images
	Feature extraction	Customized program by python *Skimage* library	Extraction of raw features of morphology and composition	Single cell features based on multiple masks
	Cell classification	Customized program by python *Sklearn* library	Cell phenotyping and identify significant marker cells	Unsupervised clustering using single cell-based features to get significant feature components. Meanwhile, cell phenotyping based on supervised methods
PM detection	PM detection of patients	Customized program by Python *Sklearn* library	Patient PM detection with feature matrix	SVM, LDA, LR models with leave-one-out cross-validation. Enable PM detection with positive probability for each patient
	Evaluation for the hybrid K-PCA algorithm, cell segmentation and classification	Customized program by Python *Sklearn*/ *roifile* library	Positive PM probability output, confusion matrix and ROC curves output comparing with conventional cytology and histopathology results	Compare the results from CY, PM and SRMC to evaluate the improvement of our SRMC method

References

1. Yue, S. et al. Cholesteryl ester accumulation induced by PTEN loss and PI3K/AKT activation underlies human prostate cancer aggressiveness. Cell Metab 19, 393–406 (2014).
